# Betulinic Acid-Enriched *Dillenia indica* L. Bark Extract Attenuates UVB-Induced Skin Aging via KEAP1-Mediated Antioxidant Pathways

**DOI:** 10.3390/antiox14091144

**Published:** 2025-09-22

**Authors:** Bo-Rim Song, Sunghwan Kim, Sang-Han Lee

**Affiliations:** 1 Department of Food Science and Biotechnology, Graduate School, Kyungpook National University, Daegu 41566, Republic of Korea; sbr9707@knu.ac.kr; 2 Department of Chemistry, Kyungpook National University, Daegu 41566, Republic of Korea; sungwhank@knu.ac.kr; 3 Mass Spectrometry Converging Research Center, and Green-Nano Materials Research Center, Daegu 41566, Republic of Korea; 4 Inner Beauty/Antiaging Center, Kyungpook National University, Daegu 41566, Republic of Korea

**Keywords:** *Dillenia indica* L., betulinic acid, KEAP1-Nrf2 signaling, UVB-induced oxidative stress, simulated gastrointestinal digestion, skin aging

## Abstract

The bark of *Dillenia indica* L. is a rich source of phenolic and triterpenoid compounds, including betulinic acid (BA), known for their antioxidant and anti-aging properties. This study investigated the antioxidant potential of a BA-enriched extract through a multidisciplinary approach combining computational, experimental, and cell-based evaluations. Molecular docking and molecular dynamics simulations revealed that BA binds stably to Kelch-like ECH-associated protein 1 (KEAP1), suggesting activation of the nuclear factor erythroid 2-related factor 2 (Nrf2) pathway. Extraction conditions were optimized using response surface methodology (RSM) and artificial neural network (ANN) modeling, yielding the maximum total phenolic content (TPC; 85.33 ± 2.26 mg gallic acid equivalents/g) and total flavonoid content (TFC; 75.60 ± 1.66 mg catechin equivalents/g), with ANN demonstrating superior predictive performance compared to RSM. Electrospray ionization tandem mass spectrometry (ESI-MS/MS) confirmed the presence of BA in the optimized extract. Simulated gastrointestinal digestion revealed reductions in TPC, TFC, and radical scavenging activity during the gastric phase. In ultraviolet B (UVB)-irradiated human keratinocyte (HaCaT) cells, the optimized extract significantly reduced intracellular reactive oxygen species (ROS) and upregulated the KEAP1-Nrf2-heme oxygenase-1 (HO-1) pathway, confirming its antioxidant mechanism. These findings highlight the extract’s stability, bioactivity, and mechanistic efficacy, supporting its application as a nutraceutical ingredient for combating oxidative stress and skin aging.

## 1. Introduction

Dietary antioxidants derived from plant sources have garnered significant attention for their potential as functional ingredients to promote health and mitigate oxidative stress-related disorders [1,2]. Among the cellular defense mechanisms, the Kelch-like ECH-associated protein 1–nuclear factor erythroid 2-related factor 2 (KEAP1-Nrf2) signaling pathway plays a central role by regulating antioxidant response elements and inducing the expression of cytoprotective enzymes such as heme oxygenase-1 (HO-1). Activation of this pathway is widely recognized as a key target in dietary strategies aimed at enhancing cellular resilience against oxidative damage [3,4,5]. Given this mechanistic framework, we hypothesized that the bioactive constituents of *Dillenia indica* L. bark could activate the KEAP1–Nrf2–HO-1 axis, thereby contributing to antioxidant and anti-aging effects.

*Dillenia indica* L. (commonly known as elephant apple) is a traditional medicinal plant consumed in several parts of Asia. Its bark is particularly rich in polyphenols and triterpenoids, including betulinic acid (BA), which have been reported to exhibit strong antioxidant and anti-aging activities [6,7,8]. However, previous studies have largely focused on general antioxidant assays or crude phytochemical analyses, without integrating computational predictions of KEAP1–ligand interactions, extraction process optimization, gastrointestinal stability assessment, and mechanistic validation in skin-relevant cell models. Accordingly, the present study aimed to bridge this gap by employing a multidisciplinary approach explicitly linking the KEAP1–Nrf2 mechanistic hypothesis to complementary computational, chemical, and biological investigations. Molecular docking and molecular dynamics simulations were employed to elucidate the interaction between BA and KEAP1, offering insights into its potential to activate the KEAP1–Nrf2–HO-1 pathway. Extraction conditions for phenolics and flavonoids were optimized using response surface methodology (RSM) and artificial neural network (ANN) modeling. Electrospray ionization tandem mass spectrometry (ESI-MS/MS) profiling further confirmed BA as a major bioactive constituent in optimized *Dillenia indica* L. bark (ODB). Additionally, the stability and bioaccessibility of ODB’s antioxidants and elastase inhibitory activities were assessed under simulated gastrointestinal conditions, an essential step in evaluating potential in vivo functionality. Finally, the antioxidant mechanism was validated in ultraviolet B (UVB)-irradiated human keratinocyte (HaCaT) cells by assessing intracellular reactive oxygen species (ROS) levels and the expression of KEAP1, Nrf2, and HO-1.

This integrated strategy combining in silico modeling, optimized extraction, advanced chemical profiling, simulated digestion, and mechanistic cell-based assays has not been previously reported for *D. indica* bark. Collectively, these findings provide robust evidence that ODB is a promising antioxidant-rich extract with stable bioactivity during digestion and strong potential for development as a nutraceutical targeting oxidative stress and skin aging.

## 2. Materials and Methods

### 2.1. Molecular Docking and Molecular Dynamics Simulations

Molecular docking and molecular dynamics (MD) simulations were performed based on previously reported protocols [9], with modifications tailored to the objectives of this study. Docking was performed using AutoDock Vina (version 1.2.7). The crystal structure of KEAP1 (PDB ID: 4L7B) was preprocessed in AutoDock Tools by removing water molecules and adding polar hydrogens. The structure of BA was retrieved from the PubChem database and energy-minimized using the MMFF94 force field in Chem3D prior to docking. The docking process in AutoDock Vina employed a grid box centered on KEAP1’s Kelch domain. Binding affinities and interacting residues were recorded from the top-scoring pose. The resulting KEAP1–ligand complexes were then subjected to 100 ns MD simulations using YASARA Structure (version 23.12.24) with the AMBER14 force field. System equilibration, energy minimization, and simulation steps followed the default YASARA protocols. Structural stability and dynamics were evaluated using root-mean-square deviation (RMSD), solvent-accessible surface area (SASA), radius of gyration (Rg), and root-mean-square fluctuation (RMSF) analyses.

### 2.2. Preparation of Sample

Bark specimens of *D. indica* L., were collected from wild trees growing on the campus of Jahangirnagar University, located in Savar, Dhaka, Bangladesh (Voucher No. 49403). The bark samples were collected in August 2019, air-dried at room temperature, mechanically ground, and stored in airtight containers at 4 °C until further use in subsequent experiments.

### 2.3. Experimental Design and Statistical Optimization via RSM

To optimize the extraction of *D. indica* bark (ODB), a central composite design (CCD) was employed using total phenolic content (TPC, Y_1_) and total flavonoid content (TFC, Y_2_) as response variables. Extraction temperature (X_1_), time (X_2_), and ethanol concentration (X_3_) were selected as independent variables. A second-order polynomial model (Equation (1)) was used to evaluate the interaction effects among these variables:

(1) $$Y=\;\beta_{0}+\sum_{i=1}^{n}\beta_{i}X_{i}+\sum_{i=1}^{n}\beta_{ii}X_{ii}^{2}+\sum_{i}^{n-1}\sum_{j}^{n}\beta_{ij}X_{ij}$$

where Y is the predicted response; Xi and Xj are coded independent variables; β_0_ is the intercept; β_i_, β_ii_, and β_ij_ are the coefficients for linear, quadratic, and interaction terms, respectively. Three-dimensional (3D) surface plots were generated to visualize these interactions.

### 2.4. Predictive Modeling of Extraction Outcomes Using ANN

To capture nonlinear relationships between extraction parameters and antioxidant yields, a multilayer perceptron (MLP) artificial neural network was employed. The input variables were extraction temperature (X_1_), extraction time (X_2_), and ethanol concentration (X_3_), while the outputs were total phenolic content (TPC, Y_1_) and total flavonoid content (TFC, Y_2_). The dataset was randomly divided into training (65%), validation (20%), and testing (15%) subsets to evaluate model generalization performance.

The ANN architecture comprised three layers: an input layer with three neurons, a hidden layer containing 10 neurons, and an output layer with one neuron for each response variable. The hidden layer utilized the hyperbolic tangent sigmoid (tansig) activation function, and the output layer employed a linear (purelin) function. Training was conducted using both feed-forward and cascade feed-forward configurations, optimized via the Broyden–Fletcher–Goldfarb–Shanno (BFGS) and Levenberg–Marquardt (trainlm) algorithms. The learning rate was set to 0.01, with weights initialized using the Nguyen–Widrow method. Early stopping was applied when the validation means squared error (MSE) failed to improve for 10 consecutive epochs.

Model performance was assessed using multiple statistical indicators, including the coefficient of determination (R^2^), root mean squared error (RMSE), average absolute deviation (AAD), and standard error of prediction (SEP), calculated according to Equations (2)–(6). The optimized ANN models achieved high R^2^ values (>0.98) across all datasets and exhibited low MSE, indicating robust predictive capability with minimal overfitting.

(2) $$MSE=\frac{1}{N}\sum_{i=1}^{N}\left(Y_{p}-Y_{e}\right)$$

(3) $$R^{2}=1-\frac{\sum_{i=1}^{n}{\left(Y_{p}-Y_{e}\right)}^{2}}{\sum_{i=1}^{n}{\left(Y_{m}-Y_{e}\right)}^{2}}$$

(4) $$RMSE=\sqrt{\frac{\sum_{i=1}^{n}{\left(Y_{p}-Y_{e}\right)}^{2}}{n}}$$

(5) $$AAD\;\left(\%\right)=\left[\frac{\sum_{i=1}^{n}\left(\frac{\left|Y_{p}-Y_{e}\right|}{Y_{e}}\right)}{n}\right]\times 100$$

(6) $$SEP\;\left(\%\right)=\frac{RMSE}{Y_{m}}\times 100$$

where Yp is the predicted response, Ye is the experimental (observed) response, Ym is the mean of experimental responses, and n is the number of observations (experiments).

### 2.5. Determination of TPC and TFC

TPC and TFC of the extracts were determined using the Folin–Ciocalteu and aluminum chloride colorimetric methods, respectively, as described previously [10]. For TPC determination, the extract was reacted with Folin–Ciocalteu reagent, Na_2_CO_3_, and deionized water (dH_2_O), and the absorbance was measured at 750 nm. Results are expressed as milligrams of gallic acid equivalents per gram of extract (mg GAE/g), based on a gallic acid calibration curve. TFC was measured by mixing the extract with NaNO_2_, AlCl_3_·6H_2_O, and NaOH, followed by measuring the absorbance at 506 nm. Values are expressed as milligrams of catechin equivalents per gram of extract (mg CE/g), based on a catechin standard curve.

### 2.6. Structural Characterization of Extract Constituents via ESI-MS/MS

Chemical profiling of the extract was carried out using negative-mode ESI-MS on a Q-Exactive Orbitrap mass spectrometer (Thermo Fisher Scientific, San Jose, CA, USA). The sample solution was directly introduced into the ESI source at a constant flow rate of 20 µL/min using a 500 µL syringe (Hamilton Company, Reno, NV, USA) operated by a syringe pump (Harvard Apparatus, Holliston, MA, USA). The instrument was configured with a mass resolution of 140,000 and scanned across a mass range of *m*/*z* 50–1000. Key instrumental parameters included a sheath gas flow rate of 5 units, no auxiliary gas, a spray voltage of 4.20 kV, a capillary temperature of 320 °C, and an automatic gain control (AGC) target of 5 × 10^6^ MS/MS was performed using normalized collision energies of 10, 20, and 30 to generate fragmentation patterns. Compound identification was accomplished by comparing observed *m*/*z* values and MS/MS fragmentation spectra with theoretical deprotonated ion masses (M−H)^−^, as well as reference data from both in-house databases and published literature [11]. All mass spectral data were analyzed using Xcalibur 3.1 software (Thermo Fisher Scientific), and chemical structures were annotated using ChemDraw Professional 15.0 (PerkinElmer, Waltham, MA, USA).

### 2.7. In Vitro Simulated Digestion

The gastrointestinal stability of ODB was assessed through in vitro simulated digestion using simulated salivary, gastric, and intestinal fluids (SSF, SGF, and SIF, respectively), following the standardized INFOGEST protocol with slight modifications [12]. SSF was prepared by dissolving 0.15 M NaCl, 3 mM urea, 2 mM KCl, and α-amylase from human saliva (Sigma-Aldrich, St. Louis, MO, USA, A1031) in deionized water and adjusting the pH to 6.8 with 1 M NaOH or HCl. SGF contained 0.2% (*w*/*v*) NaCl and 0.32% pepsin from porcine gastric mucosa (P6887, Sigma-Aldrich), with the pH adjusted to 2.0 using 1 M HCl. SIF contained 0.68% KH_2_PO_4_, 1% trypsin from porcine pancreas (T0303, Sigma-Aldrich), and 0.2% bile salts, with the pH adjusted to 7.0 using 1 M NaOH. For the digestion process, 2 mL of ODB extract (10 mg/mL) was first mixed with an equal volume (2 mL) of SSF and incubated at 37 °C for 2 min with gentle shaking, giving a total oral-phase volume of 4 mL. In accordance with the INFOGEST 1:1 ratio guideline, SGF was added at a volume equal to the total oral-phase volume (4 mL) and incubated for 2 h at 37 °C. Subsequently, SIF was added at a volume equal to the total gastric-phase volume (8 mL) and incubated for another 2 h. At each stage, aliquots were collected, immediately cooled on ice, and stored at −20 °C for subsequent antioxidant and elastase inhibitory activity assays.

### 2.8. Elastase Inhibition Assay

Elastase inhibitory activity was evaluated using porcine pancreatic elastase (0.04 U; E0258, Sigma-Aldrich) and N-succinyl-Ala-Ala-Ala-Ala-p-nitroanilide (0.78 mM; S4760, Sigma-Aldrich) as the substrate. Both enzyme and substrate were prepared in 0.1 M Tris-HCl buffer (pH 8.8). Reaction mixtures were incubated at 37 °C, and absorbance was measured at 405 nm. The percentage inhibition of elastase activity was calculated relative to an untreated control.

### 2.9. Cell-Free Antioxidant Assays

The antioxidant activities of ODB and its digested fractions were assessed using 2,2-diphenyl-1-picrylhydrazyl (DPPH), 2,2′-azino-bis(3-ethylbenzothiazoline-6-sulfonic acid) (ABTS), ferric reducing antioxidant power (FRAP), and cupric ion reducing antioxidant capacity (CUPRAC) assays, following standard protocols with minor modifications [11,13,14,15]. Ascorbic acid was used as a positive control in all assays. For each assay, the percentage of radical scavenging or reducing activity was calculated using Equation (7):

(7) $$(\%\;\mathrm{inhibition})\;=\;\left[1-\frac{{Abs}_{sample}}{{Abs}_{conrol}}\right]\times 100$$

where Abs sample and Abs control represent the absorbance of the sample and control, respectively. Each sample was tested in triplicate. DPPH and ABTS radical scavenging activities were measured at 520 nm and 595 nm, respectively. FRAP was evaluated at 520 nm, and CUPRAC at 450 nm. Antioxidant capacities were quantified using calibration curves generated with ascorbic acid, and results are expressed as μmol ascorbic acid equivalents per gram of extract (μmol ASCE/g).

### 2.10. UVB-Induced ROS Measurement and Western Blot Analysis

Intracellular ROS levels and protein expression were analyzed as previously described [16], with slight modifications. HaCaT cells (AddexBio Technologies, San Diego, CA, USA; 1 × 10^5^ cells/well) were seeded in 6-well plates and pretreated for 24 h with ODB (10, 30, and 70 μg/mL) or gallic acid (GA, 25 μM). Cells were then exposed to UVB irradiation (40 mJ/cm^2^) and analyzed for ROS using 10 μM DCF-DA (30 min incubation at 37 °C; excitation/emission 485/535 nm).

For Western blotting, cells were harvested 9 h post-UVB, lysed in RIPA buffer, and protein concentrations determined by the BSA method using the BCA protein kit (Pierce, Rockford, IL, USA). Proteins were separated by sodium dodecyl sulfate polyacrylamide gel electrophoresis (SDS-PAGE) and transferred onto polyvinylidene fluoride (PVDF) membranes. The PVDF membranes. The membranes were probed with specific primary antibodies against phosphorylated Nrf2 (p-Nrf2; Abcam, Cambridge, UK), KEAP1 (Cell Signaling Technology, Beverly, MA, USA), β-actin (Santa Cruz Biotechnology, Dallas, TX, USA), and HO-1 (Santa Cruz Biotechnology) at a dilution of 1:1000. HRP-conjugated secondary antibodies (1:5000) and enhanced chemiluminescence (ECL) reagents were used for detection.

### 2.11. HO-1 Protein Quantification Using Enzyme-Linked Immunosorbent Assay (ELISA)

Following treatment, cells were washed twice with ice-cold phosphate-buffered saline (PBS) and lysed using either the lysis buffer provided in the HO-1 ELISA Kit (ab207621; Abcam) or RIPA buffer supplemented with protease inhibitors. Lysates were collected by scraping, then centrifuged at 12,000× *g* for 10 min at 4 °C. The resulting supernatants were used for HO-1 quantification. Total protein concentrations were measured using the bicinchoninic acid (BCA) assay (Thermo Fisher Scientific) to normalize sample loading. HO-1 levels were determined according to the manufacturer’s instructions. Standards and samples (50–100 μL) were loaded into 96-well plates, incubated with the detection antibody at 37 °C for 1 h, washed, and developed with 3,3′,5,5′-tetramethylbenzidine (TMB) substrate. Absorbance was measured at 450 nm, and HO-1 concentrations were calculated using a standard curve generated from recombinant HO-1 protein (provided in the kit).

### 2.12. Statistical Analysis

Data analysis was conducted using Design Expert 11 (Stat-Ease, Minneapolis, MN, USA) for RSM, GraphPad Prism 9 (version 9.0.2; GraphPad Software, San Diego, CA, USA) for statistical comparisons, and MATLAB R2020a (Neural Network Toolbox) for ANN modeling. Results are reported as mean ± standard deviation (SD) from three independent replicates (n = 3), with statistical significance defined at *p* < 0.05, 0.01, and 0.001.

Design Expert 11 was used to assess RSM model adequacy in the context of ultrasound-assisted extraction (UAE), considering parameters such as lack of fit, R^2^, adjusted R^2^, coefficient of variation (CV), and F-value. In contrast, GraphPad Prism 9 was employed to analyze biological activity data using one-way analysis of variance (ANOVA) followed by Tukey’s multiple comparison test, with *p* < 0.05 considered statistically significant.

## 3. Results

### 3.1. Molecular Docking of BA with KEAP1

To assess the KEAP1-targeting potential of structurally related lupane-type triterpenoids, molecular docking simulations were conducted using betulin (BN), BA, and betulonic acid (BO) against the Kelch domain of human KEAP-1 (PDB ID: 4L7B). Among the three compounds, BA exhibited the strongest binding affinity to KEAP1, with a docking score of −8.3 kcal/mol, followed by BO (−8.1 kcal/mol) and BN (−7.8 kcal/mol) (Table S1). To further validate the mechanistic relevance of BA in modulating oxidative stress responses, sulforaphane (SP), a well-established Nrf2 activator, was included as a reference compound for comparative molecular docking analysis. SP is a naturally occurring isothiocyanate found in cruciferous vegetables (Brassicaceae family) [17] and is widely studied for its antioxidant, anti-inflammatory, and chemopreventive properties [18,19]. Previous studies have demonstrated that SP activates the KEAP1–NRF2 signaling pathway by covalently modifying reactive cysteine residues in KEAP1, particularly Cys151, thereby disrupting KEAP1–Nrf2 interactions and promoting Nrf2 nuclear translocation [20,21].

**Figure 1:**
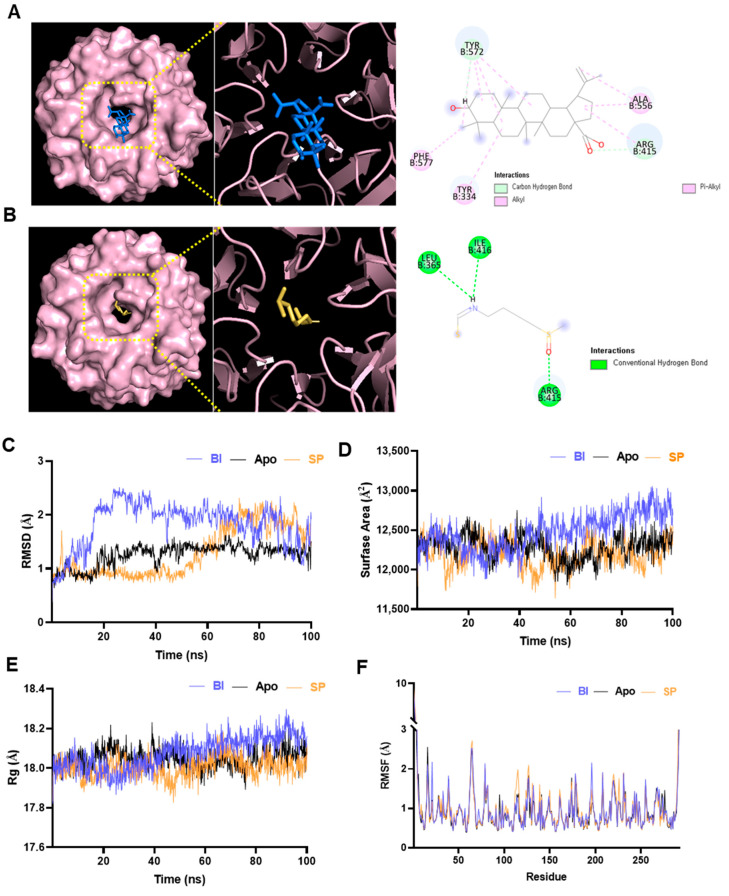
Molecular interaction and dynamics of betulinic acid (BA) and sulforaphane (SP) with Kelch-like ECH-associated protein 1 (KEAP1). (**A**) Molecular docking of BA into the Kelch domain of KEAP1, showing binding within the central pocket and interactions with key residues. (**B**) Interaction of SP with KEAP1. (**C**) Root-mean-square deviation (RMSD) plots over 100 ns; the BA–KEAP1 complex showed slightly higher RMSD, indicating conformational adaptation. (**D**) Solvent-accessible surface area (SASA) analysis revealed greater surface exposure in the BA complex. (**E**) Radius of gyration (Rg) profiles showed minor relaxation in the BA–KEAP1 complex. (**F**) Root-mean-square fluctuation (RMSF) analysis indicated increased flexibility in loop regions (~residues 50–70 and 180–200) in both BA and SP complexes.

In docking simulations with the Kelch domain of KEAP1, BA showed a significantly higher binding affinity (−8.3 kcal/mol) compared to SP (−4.5 kcal/mol), indicating a stronger and potentially more stable interaction within the KEAP1 active site. Interaction analysis revealed that BA formed multiple hydrogen bonds and hydrophobic interactions with key residues such as TYR572, ALA556, ARG415, PHE577, and TYR334, facilitating stable binding within the Kelch pocket (Figure 1A). In contrast, SP, due to its smaller and more electrophilic structure, formed fewer non-covalent interactions, consistent with its known covalent-binding mechanism (Figure 1B). These findings suggest that BA may act as a competitive or allosteric modulator of KEAP1, similar to SP, but through a distinct, non-covalent binding mechanism. The higher binding energy and broader interaction profile of BA support its potential to effectively modulate the KEAP1–Nrf2 pathway, consistent with the in vitro findings of Nrf2 activation and enhanced antioxidant responses in this study.

### 3.2. Structural Dynamics of KEAP1 Complexes via MD Simulation

Before interpreting the MD simulation results, it is essential to define the key structural descriptors used in the analysis. RMSD quantifies the temporal stability of protein–ligand complexes by measuring atomic deviations from the initial structure [22]. SASA assesses changes in the protein’s exposed surface, indicating conformational expansion or compaction upon ligand binding [23,24]. Rg reflects the overall compactness of the protein; an increase may indicate partial unfolding or relaxation of the tertiary structure. RMSF evaluates residue-level flexibility, identifying regions of increased mobility that may influence binding dynamics or functional conformational changes [25,26].

To further investigate the stability and conformational dynamics of KEAP1 upon ligand binding, 100 ns MD simulations were performed for the unbound (apo) KEAP1 protein and its complexes with BA and SP. The trajectories were analyzed using RMSD, SASA, Rg, and RMSF to assess both global and local structural changes (Figure 1C–F). RMSD analysis (Figure 1C) showed that all systems reached dynamic equilibrium around 60 ns. Between 60 and 100 ns, the BA–KEAP1 complex exhibited an average RMSD of 1.80 Å, while the SP–KEAP1 complex maintained a comparable average of 1.77 Å, indicating stable complex formation in both cases. Slightly greater RMSD fluctuations were observed in the BA complex, likely due to its larger molecular size and more extensive interaction profile. SASA analysis (Figure 1D) revealed that the BA–KEAP1 complex consistently maintained a higher SASA (~12,550 Å^2^) compared to the apo-bound (12,300 Å^2^) and SP-bound (~12,150 Å^2^) forms. This suggests that BA binding induces mild conformational expansion of the KEAP1 surface, potentially enhancing its interaction interface. Consistent with this, the Rg values (Figure 1E) showed that the BA–KEAP1 complex had a slightly higher average Rg (~18.2 Å) than the SP-bound (~17.95 Å) and apo-bound (~18.0 Å) forms. The increase in Rg reflects a more relaxed yet structurally compact conformation in the presence of the bulkier BA ligand. RMSF profiles (Figure 1F) demonstrated localized increases in flexibility in loop regions (notably residues 50–70 and 180–200) for both ligand-bound complexes. In the BA–KEAP1 complex, residues PRO384 (RMSF: 2.514 Å), ASP385 (2.527 Å), and HIS612 (3.501 Å) exhibited moderate fluctuations. In contrast, the SP–KEAP1 complex showed slightly higher fluctuations at the same residues (2.584 Å, 2.719 Å, and 4.832 Å, respectively), particularly near HIS612 within the Kelch domain. This suggests that SP binding induces greater local flexibility, whereas BA promotes more controlled structural changes.

Collectively, these findings confirm that both BA and SP form energetically stable complexes with KEAP1. However, BA induces more pronounced yet structurally regulated adjustments, aligning with its higher binding energy observed in docking studies and reinforcing its potential as a modulator of the KEAP1–Nrf2 signaling pathway.

### 3.3. Extraction Behavior and RSM-Based Optimization of Phenolic and Flavonoid Compounds from Dillenia indica L. Bark

To assess the effects of these parameters, single-factor experiments were first conducted (Figure S1). TPC (Figure S1A) increased with rising temperature, peaking at 50 °C (60.81 ± 1.28 mg GAE/g). However, a slight decline was observed at 60 °C, suggesting thermal degradation of sensitive phenolic compounds. TFC showed a similar trend, reaching a maximum at 40 °C (63.48 ± 1.06 mg CAE/g), with no significant improvement at higher temperatures (Figure S1D). Ethanol concentration significantly affected both TPC and TFC, with peak yields at 70% ethanol (TPC: 54.78 ± 0.88 mg GAE/g; TFC: 63.17 ± 0.92 mg CAE/g) (Figure S1B,E). Concentrations below 50% showed lower efficiency, likely due to poor solubility of semi-polar phenolic and flavonoid compounds. TPC steadily increased with extraction time, peaking at 30 min (52.92 ± 1.33 mg GAE/g), after which the yield plateaued (Figure S1C). Similarly, TFC (Figure S1F) rose sharply between 10 and 30 min, reaching 68.65 ± 1.22 mg CAE/g, with no significant increase observed at 40 or 50 min, indicating a potential saturation point in flavonoid extraction. These results highlight 50 °C, 70% ethanol, and 30 min as the most effective conditions for extracting phenolic and flavonoid compounds from ODB.

**Figure 2:**
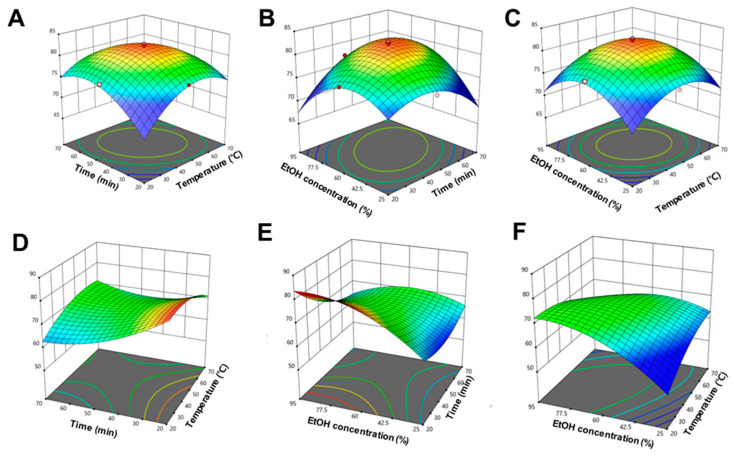
Three-dimensional response surface plots illustrating the interaction effects of extraction parameters on the total phenolic content (TPC) and total flavonoid content (TFC) obtained from *Dillenia indica* L. bark. (**A**–**C**) Effects of interactions between (**A**) temperature and time, (**B**) ethanol concentration and time, and (**C**) ethanol concentration and temperature on TPC. (**D**–**F**) Effects of interactions between (**D**) temperature and time, (**E**) ethanol concentration and time, and (**F**) ethanol concentration and temperature on TFC. All surfaces display pronounced curvature, reflecting the significant nonlinear and interactive nature of the extraction process, as modeled by the second-order polynomial equations.

To further optimize the process, RSM was applied using a second-order quadratic polynomial model. ANOVA results (Table S2) validated the statistical robustness of the TPC and TFC models. The TPC model showed excellent fit, with R^2^ = 0.9986, adjusted R^2^ = 0.9973, and predicted R^2^ = 0.9915. Among the linear terms, only extraction time (X_2_) was significant (*p* < 0.0001), while all interaction (X1X2, X1X3, X2X3) and quadratic (X12, X22, X32) terms were highly significant (*p* < 0.0001). For TFC, ethanol concentration (X3) had the most significant linear effect (*p* < 0.0001), with moderate contributions from temperature and time. All interaction and quadratic terms were also significant (*p* < 0.05). The 3D response surface plots (Figure 2A–F) visually depict these interactions. Lack-of-fit was not significant for either model (TPC: *p* = 0.2233; TFC: *p* = 0.7265), confirming model adequacy. The low coefficient of variation for TPC (0.1904%) indicated high experimental precision.

Overall, these results demonstrate the effectiveness of RSM in modeling and optimizing the extraction of phenolic and flavonoid compounds from ODB and highlight key factor interactions that enhance extraction efficiency.

### 3.4. Predictive Modeling Using ANN

**Figure 3:**
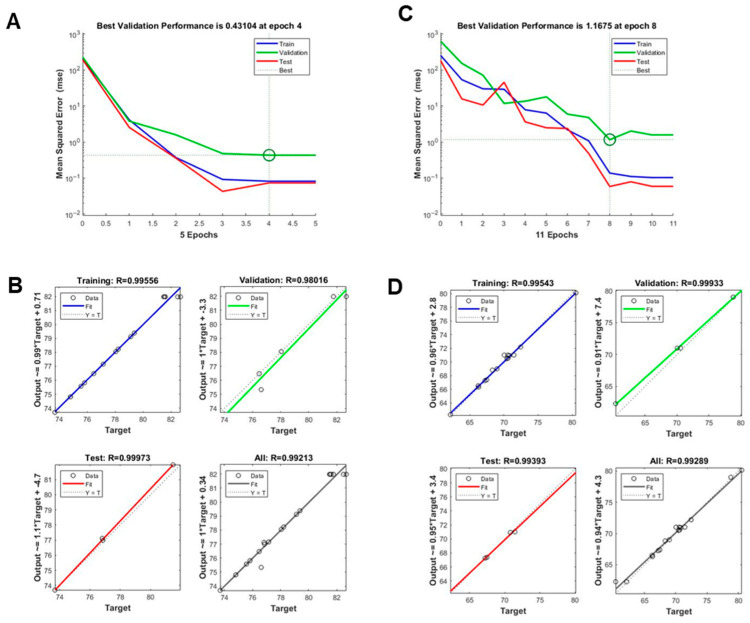
Evaluation of artificial neural network (ANN) model performance in predicting TPC and TFC. (**A**,**B**) Validation plots for the ANN model output for (**A**) TPC and (**B**) TFC. (**C**,**D**) Architecture of the optimized multilayer perceptron (MLP) model used in training, testing, and validation to minimize prediction errors for (**C**) TPC and (**D**) TFC.

ANNs have gained prominence as advanced modeling tools capable of capturing complex, nonlinear, and multidimensional relationships particularly in areas like bioengineering and extraction optimization, where traditional approaches such as RSM may have limitations [27,28]. Modeled after biological neural systems, ANNs are well-suited for identifying intricate patterns and correlations within experimental data. In this study, an ANN model was developed to predict TPC (Y1) and TFC (Y2) contents based on three extraction parameters: temperature (X1), ethanol concentration (X2), and extraction time (X3). Model performance was evaluated using standard metrics, including MSE and R^2^, across training, validation, and testing datasets. As shown in Figure 3A (TPC) and Figure 3C (TFC), the lowest validation errors were achieved at epoch 4 and epoch 8, respectively, indicating rapid convergence and absence of overfitting. The regression plots (Figure 3B,D) demonstrated strong correlations between predicted and actual values across all data partitions. The ANN model exhibited excellent predictive performance, yielded exceptionally high regression coefficients. For TPC, the R^2^ values were 0.9956 for training, 0.9945 for validation, 0.9937 for testing, and 0.9921 overall. Similarly, for TFC, the R^2^ values were 0.9954 for training, 0.9933 for both validation and testing, and 0.9930 overall.

These results confirm the high accuracy and generalizability of the ANN model. Compared to RSM, the ANN exhibited a markedly superior ability to capture complex nonlinear relationships and to predict outcomes with greater precision. This is further supported by the tight clustering of data points along the ideal prediction line in the regression plots. In summary, the ANN model effectively complemented the RSM approach, offering robust predictive capability and deeper insights into the complex extraction behavior of phenolic and flavonoid compounds from ODB.

### 3.5. Comparative Analysis of RSM and ANN Models

To evaluate and compare the predictive performance of the RSM and ANN models, various statistical parameters, including R^2^, adjusted R^2^, predicted R^2^, and lack-of-fit *p* values (Table 1; Figure 2 and Figure 3). For TPC prediction, the RSM model demonstrated high accuracy, yielding R^2^ = 0.9986, adjusted R^2^ = 0.9973, and predicted R^2^ = 0.9915, along with a non-significant lack-of-fit (*p* = 0.2233). The ANN model further improved upon this, achieving R^2^ values of 0.9956 (training), 0.9945 (validation), 0.9937 (test), and 0.9921 (overall), indicating strong predictive performance and generalizability. Similarly, for TFC prediction, the RSM model achieved R^2^ = 0.9964, adjusted R^2^ = 0.9931, and predicted R^2^ = 0.9868, with a non-significant lack-of-fit (*p* = 0.7265). The ANN model yielded comparable or slightly superior performance, with R^2^ values of 0.9954 (training), 0.9933 (validation), 0.9933 (test), and 0.9930 (overall). Although both models provided statistically significant predictions for TPC and TFC, the ANN model consistently outperformed RSM, particularly in terms of predictive accuracy and generalization. This was most evident for the TPC dataset, where the ANN closely mirrored the experimental values with minimal deviation. As summarized in Table 1, the ANN model produced predictions more closely aligned with actual experimental outcomes than RSM, reflecting its higher accuracy and robustness. While RSM remains a powerful tool for exploring variable interactions and identifying optimal conditions within a structured experimental design, ANN excels in capturing complex, nonlinear relationships and provides enhanced predictive capabilities beyond the assumptions of traditional regression models. Thus, a combined approach—utilizing RSM for experimental design and ANN for predictive modeling offers a synergistic and effective strategy for optimizing the extraction of bioactive compounds from natural sources such as *D. indica* L. bark.

Further support for this conclusion is provided in Table S2, which compares additional performance metrics, including RMSE, AAD, and SEP. The ANN model yielded lower values for all three metrics—RMSE (0.4163 for TPC, 0.3929 for TFC), AAD (0.4589% for TPC, 0.2898% for TFC), and SEP (0.0363% for TPC, 0.0359% for TFC)—thereby reinforcing its superior predictive performance over the RSM model. As presented Table S3, ANN consistently outperformed RSM in error metrics, reducing RMSE by 45% for TRC and TFC, and decreasing ADD by ~51% for TPC and 61% for TFC. Furthermore, Table S4 demonstrates that these improvements were highly reproducible across 10 random initializations, with stable R^2^ values (>0.998) and low RMSE (0.333–0.417), and paired t-tests confirmed statistically significant advantages of ANN over RSM for both TPC and TFC (*p* < 0.001).

### 3.6. ESI-MS/MS-Based Metabolite Profiling of ODB

**Figure 4:**
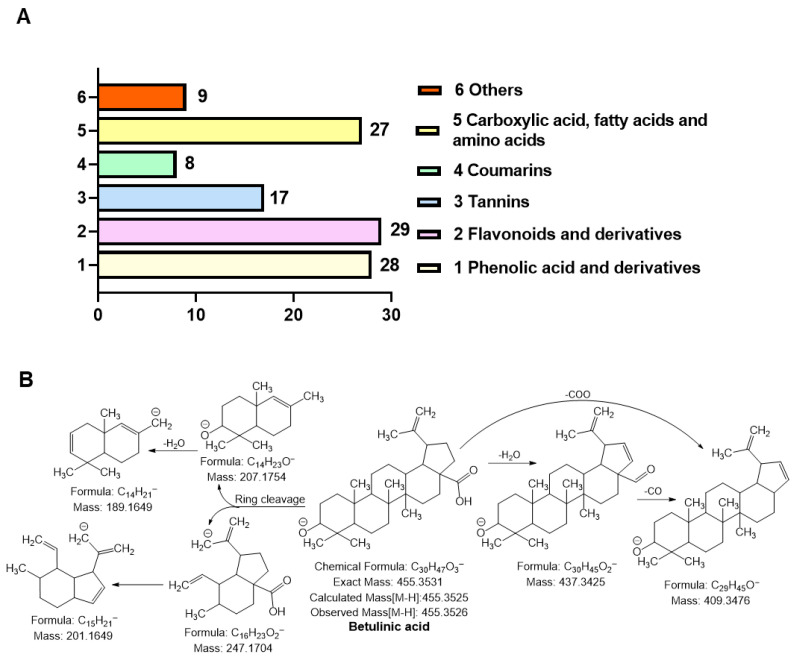
Metabolite profiling and fragmentation analysis of optimized *Dillenia indica* L. bark extract (ODB). (**A**) Classification of compounds identified via electrospray ionization tandem mass spectrometry (ESI-MS/MS), grouped into phenolic acids and derivatives, flavonoids and derivatives, tannins, coumarins, carboxylic acids/fatty acids/amino acids, and others. (**B**) Proposed MS/MS fragmentation pathway of BA, showing characteristic neutral losses and structural cleavages that confirm its identity.

To investigate the chemical constituents responsible for the bioactivities of ODB, a comprehensive metabolite profiling was conducted using high-resolution ESI-MS/MS analysis (Figure 4A). A total of 118 secondary metabolites were tentatively identified based on accurate mass measurements, retention times, and fragmentation patterns. These metabolites were categorized into major phytochemical classes, each contributing to the observed bioactivities of the extract.

Phenolic acids and derivatives (n = 28), such as gallic acid, vanillic acid, syringic acid, ferulic acid, and methyl gallate, were identified. These compounds are well-documented for their potent antioxidant and radical-scavenging properties [29]. Flavonoids and glycosides (n = 29), including naringenin, kaempferol, taxifolin, isorhamnetin, and quercetin derivatives, were also abundant. These flavonoids are known for their antioxidant, anti-inflammatory, and skin-protective properties [30]. Tannins (n = 17), such as epicatechin gallate, procyanidins A2 and B2, and catechin derivatives, were also detected and are associated with collagen stabilization and enzyme inhibitory activity relevant to skin aging [31]. Additionally, coumarins (n = 8), including umbelliferone and fraxidin, which exhibit anti-inflammatory and UVB-absorbing activities, were identified [32]. Moreover, carboxylic acids, fatty acids, and amino acid derivatives (n = 27), including ascorbic acid, malic acid, citric acid, and linoleic acid, which support skin barrier function and redox balance, were detected [33,34]. Other terpenoids and unique compounds (n = 8) identified were BA and phloridzin, which are triterpenoids with reported antioxidant and anti-aging effects [7]. A subset of these compounds was confirmed using authentic standards (denoted with # in Table S5), while several were identified for the first time in *D. indica* L. bark (marked with $). The identification of phenolic acids, flavonoids, tannins, and triterpenoids aligns well with the observed in vitro bioactivities of the optimized extract, including elastase inhibition, ROS suppression, and activation of antioxidant signaling pathways.

Table S5 provides a comprehensive overview of all identified metabolites, including their molecular formulas, observed and calculated masses, MS/MS fragment ions, and classification. These findings highlight that performing UAE under optimized conditions enhances the chemical richness and bioactive compound content of the extract. This enriched phytochemical profile provides a strong biochemical basis for its pharmacological properties and supports its potential application as a functional ingredient in skin-related therapeutics and cosmeceuticals.

### 3.7. Identification and Significance of BA

Among the bioactive triterpenoids identified in ODB, BA (C_30_H_48_O_3_) emerged as one of the most functionally significant constituents. It was detected with a precursor ion at *m*/*z* 455.3526, corresponding to the deprotonated molecular ion [M–H]^−^. The high-resolution mass spectrometric data demonstrated excellent mass accuracy, with a negligible mass deviation (Δ = 0.1 mDa) between the experimental (*m*/*z* 455.3526) and theoretical (*m*/*z* 455.3525) values, confirming the identity of BA (Table S5, Compound No. 116; Figure 4A). The MS/MS fragmentation pattern of BA revealed key diagnostic fragment ions at *m*/*z* 437.34, 409.36, and 189.16. These fragments correspond to sequential neutral losses of CO (28 Da) and H_2_O (18 Da), indicative of typical triterpenoid fragmentation behavior. In particular, the observed cleavages within the E-ring and at the C-28 carboxylic acid moiety are characteristic of lupane-type pentacyclic triterpenoids [35]. The presence of hydroxyl and carboxyl groups in BA further facilitates charge stabilization, contributing to its distinct and interpretable fragmentation pattern.

Beyond its chemical identification, BA holds significant biological relevance. It has been reported to exert potent antioxidant and anti-inflammatory effects, largely through activation of the KEAP1–Nrf2–HO-1 signaling pathway and suppression of oxidative stress mediators. Specifically, BA inhibits ROS generation and downregulates the expression of elastase and matrix metalloproteinases (MMPs), thereby contributing to its photoprotective and anti-aging activities [25,36,37]. The identification of BA in ODB not only reflects the chemical diversity achieved through the extraction process but also supports the mechanistic findings of this study. Its presence provides a molecular basis for the observed in vitro bioactivities, reinforcing the potential of ODB as a rich source of pharmacologically active compounds for dermatological and cosmeceutical applications.

### 3.8. Effect of Simulated Digestion on Antioxidant and Elastase Inhibitory Activities of ODB

**Figure 5:**
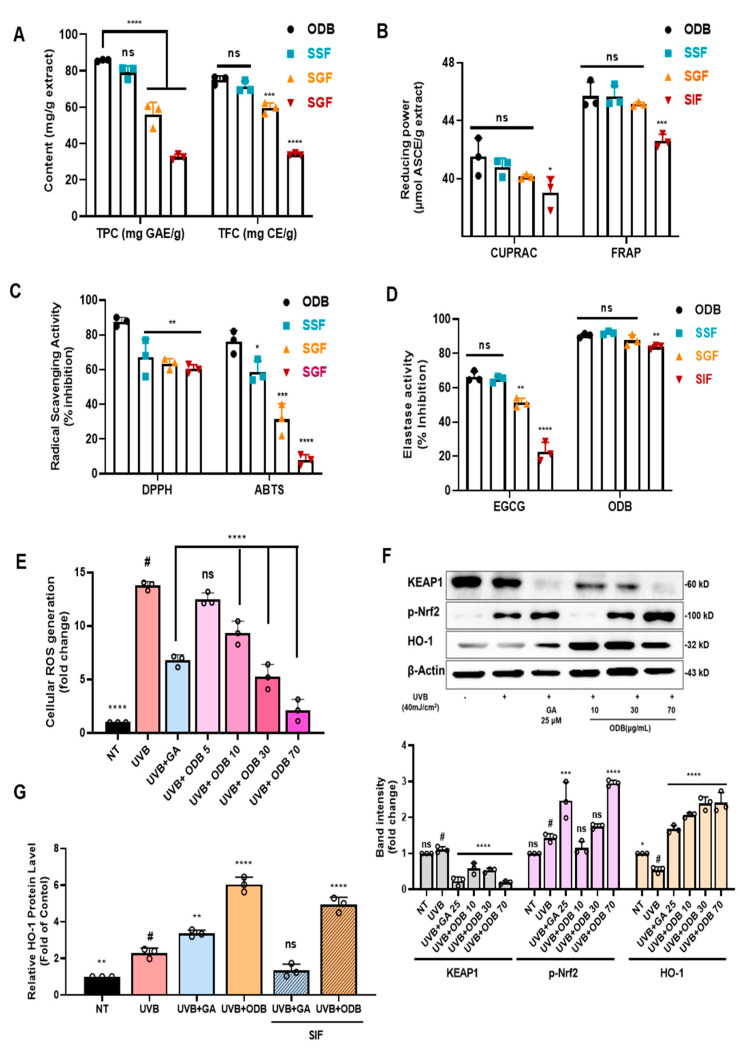
Antioxidant activity and KEAP1–nuclear factor erythroid 2–related factor 2 (Nrf2)–heme oxygenase-1 (HO-1) pathway modulation by ODB and simulated intestinal fluid-treated ODB (SIF-ODB) in UVB-irradiated HaCaT cells. (**A**–**D**) Assessment of TPC, TFC, and antioxidant activities using cupric ion reducing antioxidant capacity (CUPRAC), ferric reducing antioxidant power (FRAP), 2,2-diphenyl-1-picrylhydrazyl (DPPH), and 2,2′-azino-bis(3-ethylbenzothiazoline-6-sulfonic acid) (ABTS) assays, along with elastase inhibitory activity after simulated digestion with salivary fluid (SSF), gastric fluid (SGF), and intestinal fluid (SIF). (**E**) Intracellular reactive oxygen species (ROS) levels measured by 2′,7′-dichlorodihydrofluorescein diacetate (DCF-DA) fluorescence, indicating dose-dependent ROS reduction by ODB. (**F**) Western blot and densitometric analyses of KEAP1, phosphorylated Nrf2 (p-Nrf2), and HO-1, demonstrating ODB-induced Nrf2 pathway activation. Western blot and densitometric analyses of KEAP1, phosphorylated Nrf2 (p-Nrf2), and HO-1, demonstrating ODB-induced Nrf2 pathway activation. (**G**) HO-1 protein levels quantified by enzyme-linked immunosorbent assay (ELISA), indicating consistent induction by ODB and SIF-ODB. Effect of gallic acid (GA), ODB, and digested ODB (SIF-ODB) on HO-1 protein expression in HaCaT cells. GA was used at 100 µM as a positive control, and ODB was prepared at 10 mg/mL prior to digestion following the INFOGEST protocol. SIF-ODB refers to the supernatant obtained after simulated intestinal digestion (SIF phase), freezing, thawing, and centrifugation. Data represent mean ± standard deviation (SD), n = 3. Statistical significance: # *p* < 0.05, * *p* < 0.05, ** *p* < 0.01, *** *p* < 0.001, **** *p* < 0.0001 vs. non-treated (NT) or UVB-exposed (ODB); ns, not significant.

The stability of the antioxidant and anti-aging properties of ODB was evaluated under simulated gastrointestinal conditions, including salivary (SSF), gastric (SGF), and intestinal (SIF) phases (Figure 5A–D). SSF mimics the oral environment (pH ~7.0), SSG simulates the acidic conditions of the stomach (pH ~2.0), and SIF replicates the slightly alkaline environment of the small intestine (pH ~7.0), collectively modeling the sequential physiological digestion process [12,38].

As shown in Figure 5A, TPC and TFC were significantly affected by simulated digestion. The ODB exhibited TPC and TFC values of 85.79 ± 1.61mg GAE/g and 74.93 ± 1.22 mg CE/g, respectively. Following SGF treatment, these values decreased markedly to 56.07 ± 2.41 mg GAE/g (TPC) and 59.53 ± 2.76 mg CE/g (TFC), likely due to the structural degradation of phenolics under acidic conditions. No substantial recovery was observed in the SIF phase, with TPC and TFC remaining at reduced levels (32.63 ± 1.47 mg GAE/g and 35.02 ± 0.73 mg CE/g, respectively), indicating sustained degradation or poor solubility post-gastric transition. Figure 5B presents the results of reducing power assays (CUPRAC and FRAP), expressed in micromolar ascorbic acid equivalents per gram (μM ASCE/g). In ODB, CUPRAC and FRAP values were 42.1 ± 0.74 and 45.7 ± 2.03 μM ASCE/g, respectively. SSF-treated samples maintained comparable values, demonstrating stability during the oral phase. However, SGF treatment significantly reduced CUPRAC and FRAP values to 40.1 ± 1.45 and 45.1 ± 0.68 μM ASCE/g, respectively, aligning with the decline in TPC and TFC. As shown in Figure 5C, radical scavenging activities measured by DPPH and ABTS assays also declined substantially following SGF treatment, with inhibition dropping below 50% relative to the ODB group. This highlights the sensitivity of free radical-scavenging compounds to acidic gastric conditions.

Figure 5D illustrates changes in elastase inhibitory activity across digestion phases. Both the untreated extract and SSF-treated samples retained high elastase inhibition (~90% inhibition). Notably, following SIF treatment, ODB still maintained ~80% of its original elastase inhibitory activity, despite prior gastric degradation. This contrasts with the positive control epigallocatechin gallate (EGCG), a single-compound flavonoid known for its poor stability during digestion, which showed rapid loss of activity. The superior retention in ODB may be attributed to the synergistic effects and protective matrix formed by the complex phytochemical mixture and plant-derived fibers, which likely shield bioactive compounds during digestion. These results demonstrate that gastric conditions markedly reduced phenolic content (TPC reduced to ~38% and TFC to ~42% of the undigested extract) and diminished antioxidant capacity, while intestinal digestion did not restore these losses. Nevertheless, elastase inhibitory activity remained relatively preserved during digestion, indicating that ODB retains meaningful bioactivity even under simulated gastrointestinal conditions, supporting its potential as a stable oral nutraceutical.

### 3.9. Antioxidant Mechanism Through Nrf2 Activation and ROS Suppression

The antioxidant potential and underlying mechanism of ODB were investigated in UVB-irradiated HaCaT cells. As shown in Figure 5E, UVB exposure significantly elevated intracellular ROS levels compared to the non-treated control (NT; *p* < 0.0001). Pretreatment with ODB markedly suppressed UVB-induced ROS production in a concentration-dependent manner, with 70 μg/mL ODB reducing ROS levels nearly to those of the NT group (*p* < 0.0001). This was comparable to the effect observed with the positive control, gallic acid (GA, 25 μM). Western blot analysis (Figure 5F) revealed that UVB irradiation upregulated KEAP1 expression and downregulated p-Nrf2 and HO-1 protein levels, indicating suppression of the cellular antioxidant defense pathway. ODB pretreatment effectively reversed these changes in a dose-dependent manner: KEAP1 expression decreased significantly, while p-Nrf2 and HO-1 levels increased compared to UVB treatment alone (*p* < 0.0001). Densitometric analysis confirmed significant modulation of these key markers, demonstrating that ODB activates the KEAP1–Nrf2–HO-1 pathway. These findings were further supported by ELISA-based quantification of HO-1 protein levels (Figure 5G), which showed that UVB exposure reduced HO-1 expression compared to NT, while ODB pretreatment significantly restored it (*p* < 0.0001). Notably, SIF-treated ODB also retained the ability to upregulate HO-1 following UVB exposure, suggesting that its antioxidant efficacy is largely preserved post-digestion.

These results indicate that ODB effectively activates the KEAP1–Nrf2–HO-1 signaling axis and suppresses UVB-induced ROS generation. Furthermore, its ability to retain antioxidant function after gastrointestinal simulation highlights its promise as a stable oral antioxidant for skin protection.

## 4. Discussion

The KEAP1–Nrf2–HO-1 signaling pathway plays a pivotal role in cellular defense against oxidative stress. Under homeostatic conditions, KEAP1 binds to Nrf2, facilitating its proteasomal degradation and maintaining redox equilibrium [39]. Upon exposure to oxidative or electrophilic stress, this interaction is disrupted, allowing Nrf2 to translocate into the nucleus and promote the transcription of antioxidant and cytoprotective genes, including HO-1. HO-1 catalyzes the degradation of heme into biliverdin, carbon monoxide, and free iron, thereby exerting potent antioxidant and anti-inflammatory effects. Consequently, activation of the KEAP1–Nrf2–HO-1 axis represents a promising strategy for enhancing cellular antioxidant capacity and mitigating oxidative damage [3,40].

In this study, we comprehensively investigated the optimized extraction, gastrointestinal stability, antioxidant capacity, and mechanistic action of *Dillenia indica* L. bark extract (ODB) through an integrated approach combining bioinformatics, in vitro assays, and analytical profiling. In silico molecular docking revealed that betulinic acid (BA), a major triterpenoid identified in ODB, forms stable interactions with critical residues in the KEAP1 Kelch domain via hydrogen bonding and hydrophobic contacts. Molecular dynamics simulations over 100 ns further confirmed the stability of the BA–KEAP1 complex, accompanied by increased SASA and loop flexibility characteristics consistent with potential disruption of KEAP1–Nrf2 interactions (Figure 1). These findings underscore the utility of computational approaches for rapidly screening natural product libraries and predicting KEAP1 inhibitory potential.

Optimizing extraction parameters is crucial for maximizing the yield and stability of bioactive constituents. Response surface methodology (RSM) enables efficient modeling of linear, quadratic, and interaction effects among multiple extraction variables, while artificial neural networks (ANNs) provide robust predictive capabilities for complex, nonlinear patterns [41,42]. RSM is a statistical and mathematical tool used to explore the relationships between multiple experimental variables and one or more response variables. By fitting a second-order polynomial model, RSM identifies optimal conditions while minimizing the number of required experiments. This approach is particularly useful in extraction studies, as it allows for the evaluation of both linear and interactive effects of variables such as temperature, solvent concentration, and extraction time [43,44].

ODB contains several bioactive compounds, including BA, a pentacyclic triterpenoid known for its antioxidant, anti-diabetic, and anti-inflammatory properties [45]. Given its pharmacological potential and prevalence in ODB, optimizing extraction conditions to maximize its recovery along with other phenolic and flavonoid constituents is of significant interest. Parameters such as temperature, time, and solvent concentration are known to critically influence extraction efficiency [46]. By considering the combined effects of temperature, ethanol concentration, and extraction time, we identified optimal conditions (46.2 °C, 63.0% ethanol, 48.7 min) that maximized TPC and TFC yields with high predictive accuracy (Figure 2 and Figure 3). These parameters reflect a balanced extraction strategy: moderate temperatures enhance solute diffusion without degrading thermolabile phenolics; ethanol concentration modulates solvent polarity to solubilize both polar and semi-polar compounds; and controlled extraction time allows efficient recovery while minimizing oxidation or degradation [47,48].

Supporting these results, ESI-MS/MS profiling of ODB under optimized conditions revealed a broad spectrum of phenolic acids, flavonoids, and triterpenoids, including a prominent peak at *m*/*z* 455.35 [M–H]^−^ corresponding to BA. Its MS/MS fragmentation generated characteristic ions at *m*/*z* 437, 409, and 367, consistent with reported fragmentation patterns (Table S5), confirming the structural identity and integrity of BA within the extract. The overall mass spectral profile highlighted the richness and diversity of phenolic and flavonoid constituents, suggesting potential synergistic contributions to the extract’s antioxidant and anti-aging activities. While this study focused on qualitative high-resolution profiling to map the metabolite landscape of ODB, we recognize the importance of comprehensive quantitative validation. Future work will therefore be directed toward establishing full validation parameters (LOD, LOQ, repeatability) for key targeted analytes, beginning with BA, to enable absolute quantification and enhance analytical robustness.

Evaluating the stability of these compounds under simulated gastrointestinal conditions is essential to predict their functional efficacy following oral administration [49]. In vitro simulated digestion, which sequentially mimics the salivary (SSF), gastric (SGF), and intestinal (SIF) phases, showed significant decreases in radical scavenging activity during the acidic gastric phase, with levels remaining lower than those of the undigested extract in the subsequent intestinal phase (Figure 5B,C). These findings indicate that a substantial proportion of ODB’s bioactives withstand gastrointestinal transit, supporting their potential bioaccessibility. Compared with single isolated compounds such as EGCG and gallic acid, the complex phytochemical matrix of ODB likely provides a protective microenvironment, stabilizing key bioactives through physical entrapment and intermolecular interactions.

Post-digestion antioxidant assays (DPPH, ABTS, FRAP, and CUPRAC) confirmed the retention of considerable radical scavenging and reducing capacity (Figure 5B,C), while elastase inhibition assays demonstrated preserved anti-elastase activity (Figure 5D). Notably, the elastase inhibitory activity of ODB remained at approximately 80% even after simulated intestinal digestion, in contrast to the substantial losses observed in total phenolic/flavonoid content and radical scavenging assays. This suggests that elastase inhibition in ODB is not solely attributable to phenolics but may also involve digestion-stable constituents, such as BA, which has been reported to exhibit strong anti-elastase activity and relative stability under gastrointestinal-like conditions. Furthermore, the synergistic interplay between multiple bioactive compounds potentially including triterpenoids, residual bound phenolics, and other secondary metabolites may contribute to the preserved activity [50]. Similar synergistic effects have been documented in plant extracts where polyphenols interact with non-phenolic components to enhance bioactivity despite partial degradation of individual compounds during digestion [51]. These results collectively indicate that ODB may help maintain extracellular matrix integrity and skin elasticity, both of which are critical for mitigating visible signs of skin aging. While this study did not perform post-digestion compositional profiling to confirm the specific contributors to the retained activity, this represents a promising avenue for future research involving targeted metabolite profiling and activity-guided fractionation to identify the digestion-stable bioactives responsible for elastase inhibition.

In UVB-irradiated HaCaT keratinocytes, ODB pretreatment significantly reduced intracellular ROS levels. UVB exposure is known to induce excessive ROS production, triggering oxidative damage, activation of matrix metalloproteinases (MMPs), and degradation of collagen and elastin—hallmarks of photoaging [52,53]. Strengthening antioxidant defenses in keratinocytes is therefore essential for preventing UVB-induced skin damage. Western blot and HO-1 ELISA analyses confirmed that ODB increased p-Nrf2 and HO-1 expression while reducing KEAP1 levels, indicating activation of the KEAP1–Nrf2–HO-1 pathway (Figure 5F,G). These experimental results are consistent with the in silico predictions and suggest that BA, while not tested in isolation in cell assays, is likely a major contributor to the extract’s observed cytoprotective effects, in combination with other synergistic phytochemicals. However, it should be acknowledged that direct treatment of HaCaT keratinocytes with either undigested or digested ODB in vitro dose not fully recapitulate the physiological conditions following oral ingestion. In vivo, only bioavailable metabolites such as polyphenols, flavonoids, and triterpenoids are likely to reach the skin after gastrointestinal digestion and systemic absorption. Therefore, our findings should be interpreted as evidence of the nutraceutical potential of ODB within a controlled cellular model, rather than as conclusive proof of its efficacy in humans. Additional investigations, including evaluation of isolated TPC/TFC fractions, direct testing of BA, and co-culture systems integrating intestinal and skin cells, would provide more physiologically relevant insights into the skin-protective effects of orally ingested ODB.

Taken together, this study demonstrates that ODB optimized through RSM and validated by ANN and in silico modeling possesses a rich diversity of phenolic and flavonoid compounds as confirmed by ESI-MS/MS analysis, retains substantial antioxidant and anti-aging activity after simulated digestion, and effectively activates the KEAP1–Nrf2–HO-1 signaling axis. These findings support the nutraceutical potential of ODB as a stable, orally administered candidate for combating oxidative stress and promoting skin health.

## 5. Conclusions

This study systematically evaluated the antioxidant and anti-aging potential of ODB, enriched with BA, through an integrated approach that combined in silico modeling, extraction optimization, metabolite profiling, and biological assays. Molecular docking and MD simulations confirmed the strong and stable binding of BA to the KEAP1 Kelch domain, suggesting disruption of the KEAP1–Nrf2 interaction as a plausible mechanism for antioxidant activation. Extraction optimization using RSM identified a 63.0% ethanol concentration, 48.7 min extraction time, and 46.2 °C temperature as optimal conditions for maximizing phenolic and flavonoid yields. ANN modeling demonstrated superior predictive accuracy, capturing complex nonlinear relationships in the extraction process. ESI-MS/MS profiling validated the presence of diverse bioactive compounds, with BA identified as a major component linked to observed bioactivities. Simulated digestion revealed significant losses in TPC, TFC, and radical scavenging activity during the gastric phase, but notable recovery in the intestinal phase, indicating functional stability and potential bioaccessibility. Post-digestion, ODB retained substantial antioxidant and elastase inhibitory activities. In UVB-irradiated HaCaT cells, ODB significantly suppressed ROS levels and activated the KEAP1–Nrf2–HO-1 signaling pathway, confirming its intracellular antioxidant mechanism.

Taken together, these findings support ODB as a promising and stable oral nutraceutical candidate for mitigating oxidative stress and skin aging. However, further in vivo and clinical studies are warranted to establish its bioavailability, efficacy, and interactions with gut microbiota.

## Figures and Tables

**Table 1:** Comparison of predicted and experimental values of TPC and TFC under various UAE conditions. Predictions obtained using RSM and ANN are presented alongside experimentally measured values. Temperature (X_1_), Time (X_2_), and Ethanol Concentration (X_3_) are used as independent variables. Values are denoted as mean ± SD (n = 3).

Run	Independent Variables			Response					
	Temp (°C) (X_1_)	Time (min) (X_2_)	EC (%) (X_3_)	TPC (mg GAE/g) (Y_1_)			TFC (mg CE/g) (Y_2_)		
				RSM (pred)	ANN (pred)	Experimental	RSM (pred)	ANN (pred)	Experimental
1	45	45	95	76.59	77.15	77.15 ± 0.85	63.53	65.56	63.62 ± 1.05
2	60	30	80	76.19	75.57	75.57 ± 0.47	60.11	62.20	60.25 ± 0.22
3	20	45	60	76.05	75.34	76.62 ± 3.09	65.63	64.00	65.99 ± 1.01
4	60	30	40	78.25	79.38	79.38 ± 0.79	62.02	62.81	62.31 ± 0.25
5	30	60	40	76.00	77.11	76.82 ± 1.88	60.36	61.27	60.16 ± 0.19
6	30	60	80	79.16	78.23	78.23 ± 0.86	65.83	65.38	65.47 ± 0.49
7	60	60	80	78.32	79.13	79.13 ± 2.01	63.80	62.27	63.82 ± 1.00
8	45	45	60	81.92	81.98	81.77 ± 4.34	73.59	73.02	73.01 ± 0.56
9	45	45	60	81.92	81.98	81.53 ± 4.52	73.59	73.02	73.57 ± 1.01
10	60	60	40	75.56	75.82	75.82 ± 2.20	67.06	67.94	67.01 ± 0.19
11	45	20	60	76.28	76.47	76.48 ± 1.83	63.79	63.15	63.55 ± 1.06
12	30	30	40	75.42	74.81	74.81 ± 1.50	61.37	62.31	61.30 ± 0.15
13	70	45	60	77.71	76.99	76.86 ± 1.74	64.48	63.51	64.23 ± 0.39
14	45	45	60	81.92	81.98	82.68 ± 0.41	73.59	73.02	73.47 ± 0.94
15	45	45	60	81.92	81.98	81.49 ± 1.66	73.59	73.02	73.74 ± 1.02
16	45	45	60	81.92	81.98	82.48 ± 0.42	73.59	73.02	73.52 ± 1.00
17	45	45	25	75.63	74.81	74.80 ± 0.18	60.40	61.28	60.40 ± 0.09
18	30	30	80	73.76	73.69	73.69 ± 2.11	68.20	69.01	68.20 ± 1.09
19	45	45	60	81.92	81.98	81.61 ± 0.57	73.59	73.02	73.47 ± 0.99
20	45	70	60	78.53	78.05	78.05 ± 1.39	66.01	66.49	66.35 ± 0.49

X_1_: temperature (°C); X_2_: time (min); X_3_: ethanol concentration (%); TPC: total phenolic content (mg gallic acid equivalent/g dry weigh extract); TFC: total flavonoid content (mg catechin equivalent/g dry weigh extract).

## Data Availability

Data is contained within the article or Supplementary Materials.

## Supplementary Materials

The following supporting information can be downloaded at: https://www.mdpi.com/article/10.3390/antiox14091144/s1, Figure S1. Effect of single extraction parameters on total phenolic content (TPC; A–C) and total flavonoid content (TFC; D–F); Table S1. Binding affinity values (kcal/mol) of sulforaphane and triterpenoids (betulin, betulinic acid, and betulonic acid) with the KEAP1 Kelch domain predicted using AutoDock and MolDock docking algorithms; Table S2. ANOVA for quadratic model (function: none); Table S3. Comparison of the prediction ability of RSM and ANN. Table S4. Reproducibility and comparative performance of ANN models across multiple random initializations (n = 10 runs). Table S5. List of possible identified compounds of ODB in negative ion mode.

## Abbreviations

ANN	Artificial Neural Network
ABTS	2′-Azino-bis(3-ethylbenzothiazoline-6-sulfonic acid)
CUPRAC	Cupric Ion Reducing Antioxidant Capacity
DCF-DA	2′,7′-Dichlorofluorescin diacetate
DPPH	2,2-Diphenyl-1-picrylhydrazyl
EGCG	Epigallocatechin gallate
ELISA	Enzyme-Linked Immunosorbent Assay
ESI-MS/MS	Electrospray Ionization Tandem Mass Spectrometry
FRAP	Ferric Reducing Antioxidant Power
HO-1	Heme Oxygenase-1
HRP	Horseradish Peroxidase
KEAP1	Kelch-like ECH-Associated Protein 1
MD	Molecular Dynamics
ODB	Optimized *Dillenia indica* L. Bark extract
p-Nrf2	Phosphorylated Nuclear factor erythroid 2–related factor 2
Rg	Radius of gyration
RMSD	Root Mean Square Deviation
RMSF	Root Mean Square Fluctuation
ROS	Reactive Oxygen Species
RSM	Response Surface Methodology
SASA	Solvent-Accessible Surface Area
SGF	Simulated Gastric Fluid
SIF	Simulated Intestinal Fluid
SSF	Simulated Salivary Fluid
TFC	Total Flavonoid Content
TPC	Total Phenolic Content
UVB	Ultraviolet B
