# Supplementary material for: Betulinic Acid-Enriched Dillenia indica L. Bark Extract Attenuates UVB-Induced Skin Aging via KEAP1-Mediated Antioxidant Pathways
Source: Antioxidants (Basel). 2025 Sep 22;14(9):1144. doi: 10.3390/antiox14091144 (PMC12466404; doi:10.3390/antiox14091144)
Supplement: Supplementary file 1 [file antioxidants-14-01144-s001.zip › Supplementary Data0827.pptx]

## Slide 1
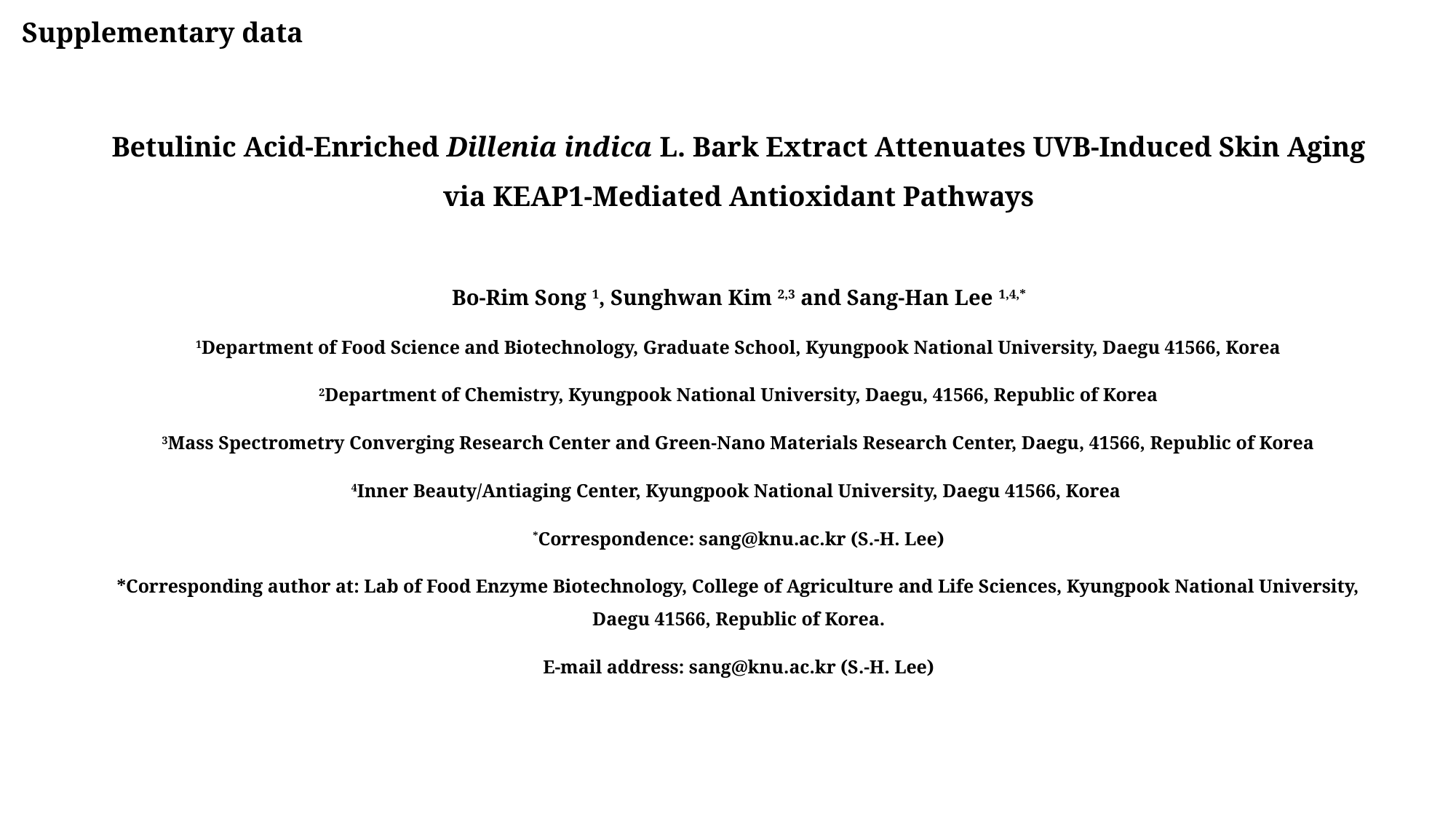

Supplementary data
Betulinic Acid-Enriched Dillenia indica L. Bark Extract Attenuates UVB-Induced Skin Aging via KEAP1-Mediated Antioxidant Pathways
Bo-Rim Song 1, Sunghwan Kim 2,3 and Sang-Han Lee 1,4,*
1Department of Food Science and Biotechnology, Graduate School, Kyungpook National University, Daegu 41566, Korea
2Department of Chemistry, Kyungpook National University, Daegu, 41566, Republic of Korea
3Mass Spectrometry Converging Research Center and Green-Nano Materials Research Center, Daegu, 41566, Republic of Korea
4Inner Beauty/Antiaging Center, Kyungpook National University, Daegu 41566, Korea
*Correspondence: sang@knu.ac.kr (S.-H. Lee)
*Corresponding author at: Lab of Food Enzyme Biotechnology, College of Agriculture and Life Sciences, Kyungpook National University, Daegu 41566, Republic of Korea.
E-mail address: sang@knu.ac.kr (S.-H. Lee)

## Slide 2
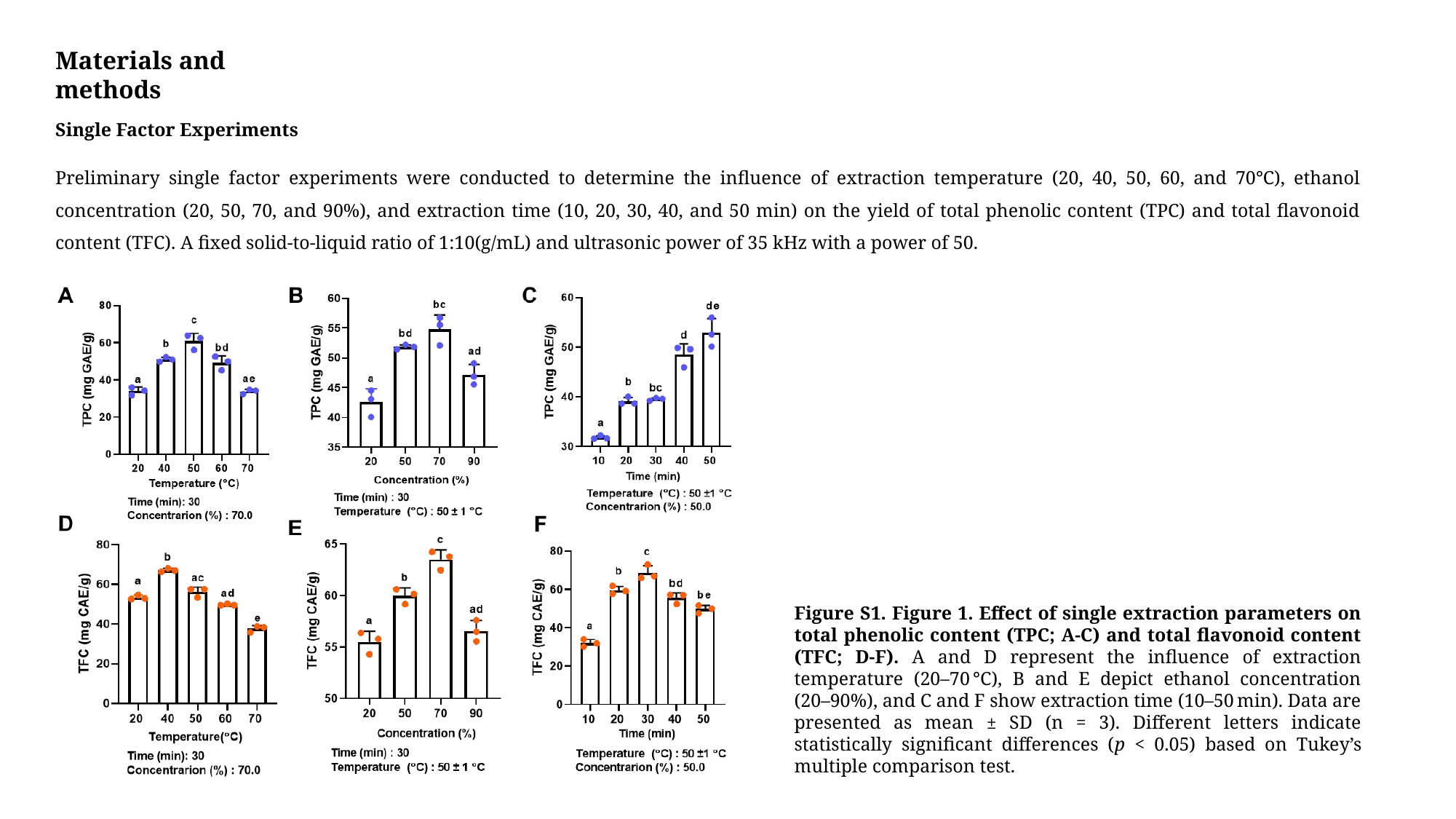

Materials and methods
Single Factor Experiments
Preliminary single factor experiments were conducted to determine the influence of extraction temperature (20, 40, 50, 60, and 70℃), ethanol concentration (20, 50, 70, and 90%), and extraction time (10, 20, 30, 40, and 50 min) on the yield of total phenolic content (TPC) and total flavonoid content (TFC). A fixed solid-to-liquid ratio of 1:10(g/mL) and ultrasonic power of 35 kHz with a power of 50.
Figure S1. Figure 1. Effect of single extraction parameters on total phenolic content (TPC; A-C) and total flavonoid content (TFC; D-F). A and D represent the influence of extraction temperature (20–70 °C), B and E depict ethanol concentration (20–90%), and C and F show extraction time (10–50 min). Data are presented as mean ± SD (n = 3). Different letters indicate statistically significant differences (p < 0.05) based on Tukey’s multiple comparison test.

## Slide 3
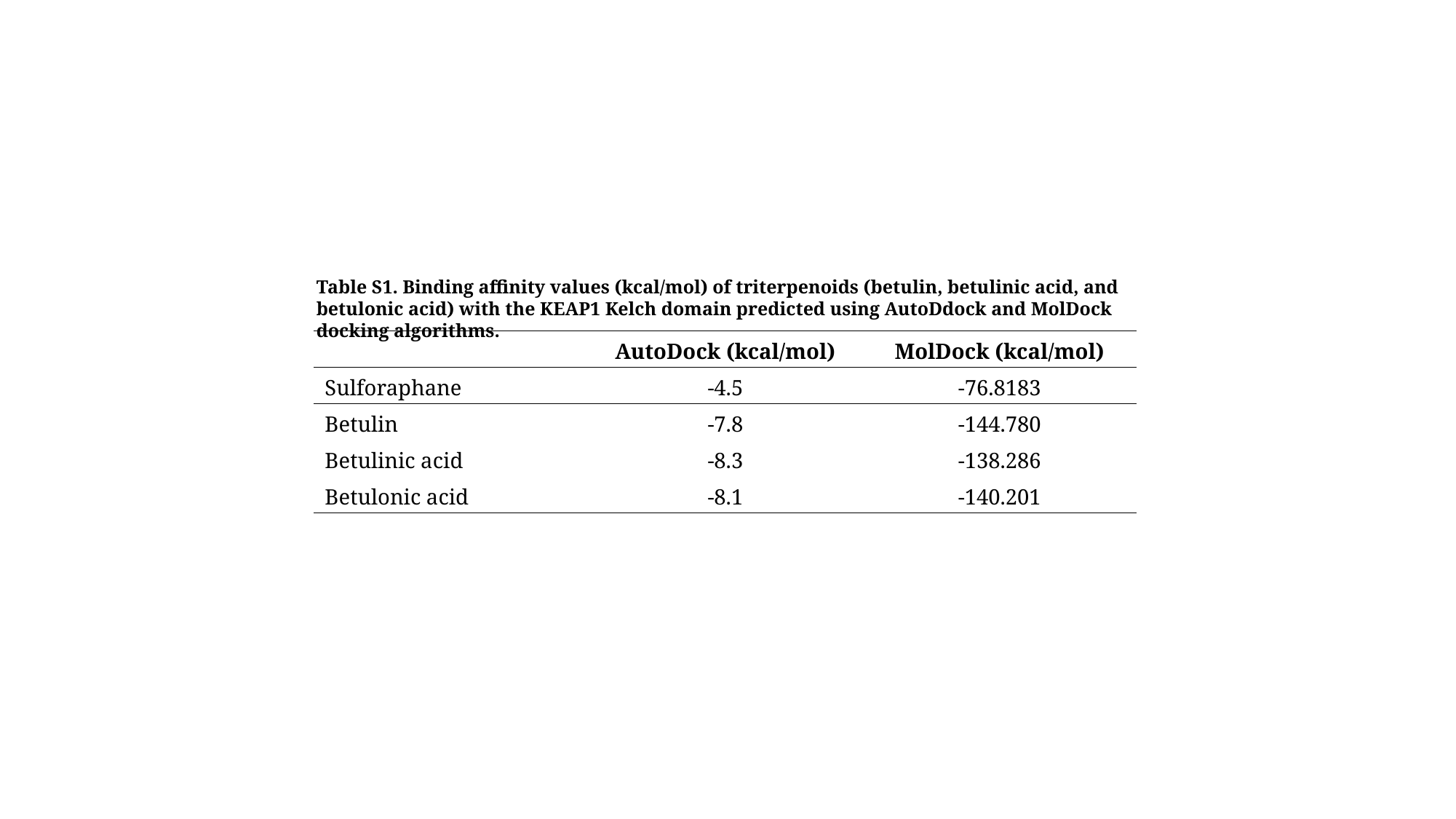

Table S1. Binding affinity values (kcal/mol) of triterpenoids (betulin, betulinic acid, and betulonic acid) with the KEAP1 Kelch domain predicted using AutoDdock and MolDock docking algorithms.
| | AutoDock (kcal/mol) | MolDock (kcal/mol) |
| --- | --- | --- |
| Sulforaphane | -4.5 | -76.8183 |
| Betulin | -7.8 | -144.780 |
| Betulinic acid | -8.3 | -138.286 |
| Betulonic acid | -8.1 | -140.201 |

## Slide 4
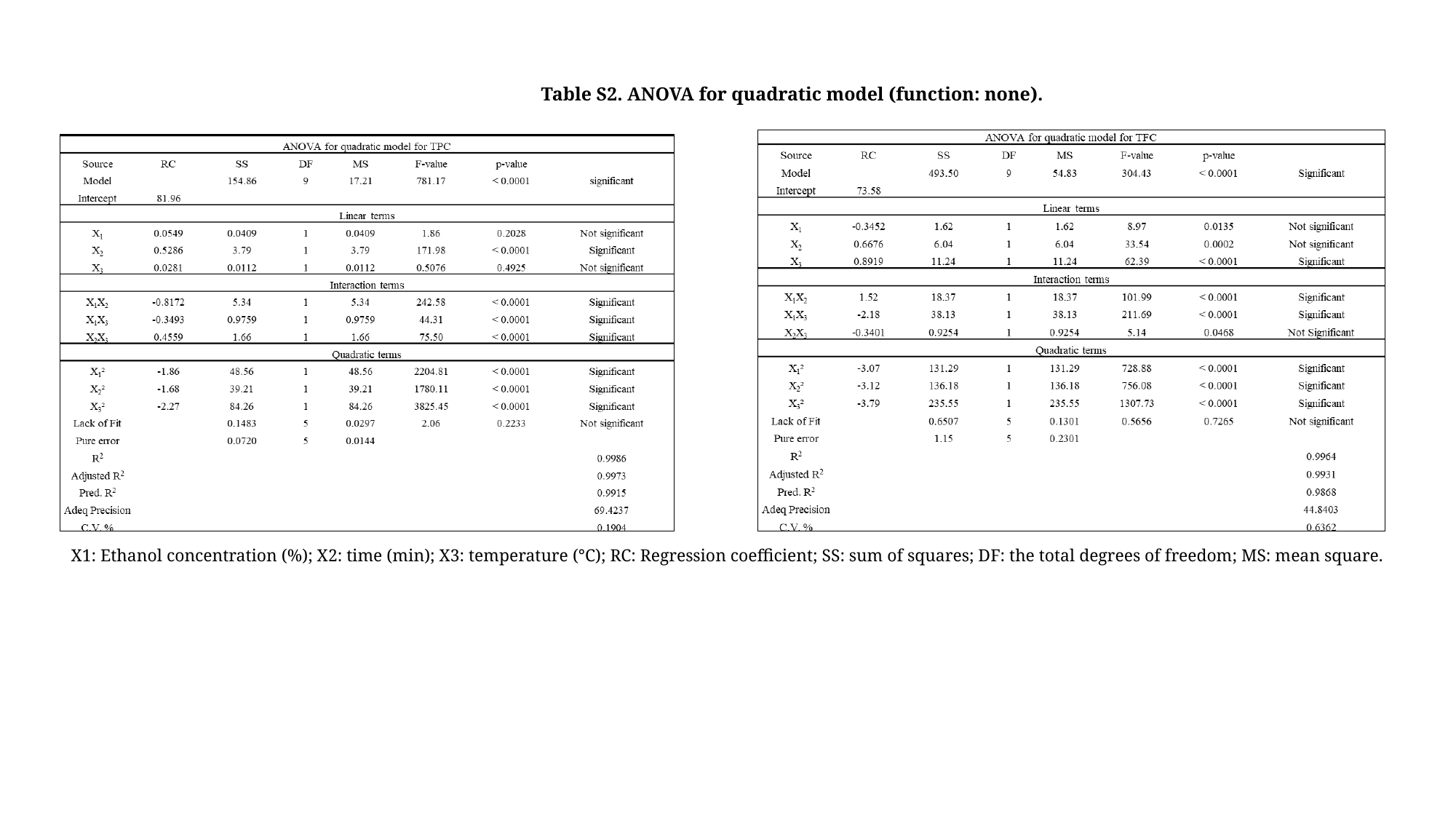

Table S2. ANOVA for quadratic model (function: none).
X1: Ethanol concentration (%); X2: time (min); X3: temperature (°C); RC: Regression coefficient; SS: sum of squares; DF: the total degrees of freedom; MS: mean square.

## Slide 5
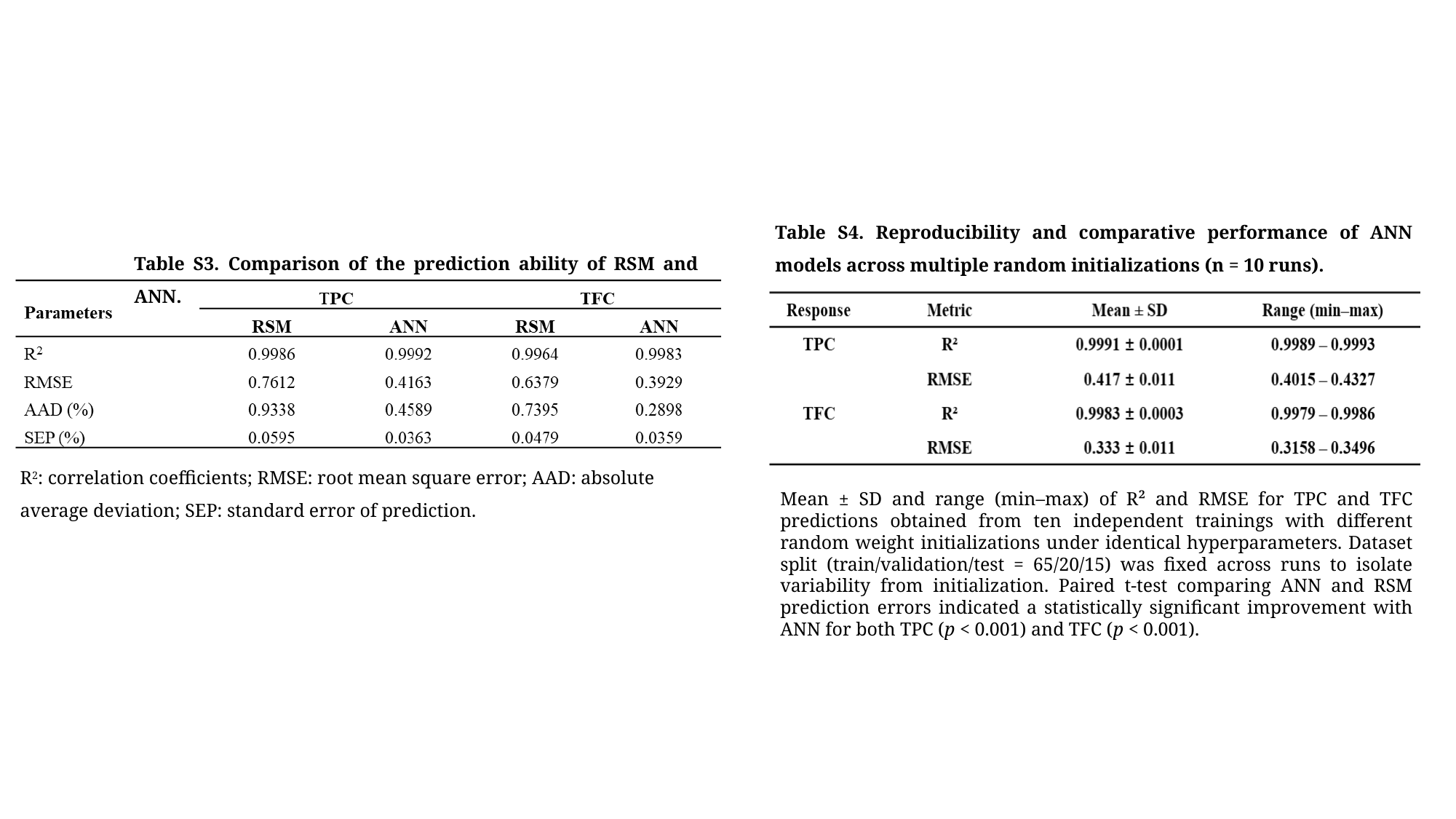

Table S4. Reproducibility and comparative performance of ANN models across multiple random initializations (n = 10 runs).
Table S3. Comparison of the prediction ability of RSM and ANN.
R2: correlation coefficients; RMSE: root mean square error; AAD: absolute average deviation; SEP: standard error of prediction.
Mean ± SD and range (min–max) of R² and RMSE for TPC and TFC predictions obtained from ten independent trainings with different random weight initializations under identical hyperparameters. Dataset split (train/validation/test = 65/20/15) was fixed across runs to isolate variability from initialization. Paired t-test comparing ANN and RSM prediction errors indicated a statistically significant improvement with ANN for both TPC (p < 0.001) and TFC (p < 0.001).
