# Supplementary material for: Betulinic Acid-Enriched Dillenia indica L. Bark Extract Attenuates UVB-Induced Skin Aging via KEAP1-Mediated Antioxidant Pathways
Source: Antioxidants (Basel). 2025 Sep 22;14(9):1144. doi: 10.3390/antiox14091144 (PMC12466404; doi:10.3390/antiox14091144)
Supplement: Supplementary file 1 [file antioxidants-14-01144-s001.zip › Table.pdf]

Table S5. List of possible identified compounds of ODB in negative ion mode.

	No.	Compound name	EF	OM (*m/z*)	CM (*m/z*)	MS/MS	CL
Phenolic acids and derivatives	1	Phloroglucinol	$C_6H_6O_3$	125.0227	125.0238	97.03, 95.01, 83.04, 79.01	2
	2	Salicylic acid[#]	$C_7H_6O_3$	137.0236	137.0238	119.02, 109.01, 93.03	1
	3	Ethyl benzoate[#]	$C_9H_{10}O_2$	149.0608	149.0603	121.02, 103.01	1
	4	Protocatechuic acid[#]	$C_7H_6O_4$	153.0186	153.0187	135.00, 109.02	1
	5	Coumaric acid[#]	$C_9H_8O_3$	163.0394	163.0395	145.02, 119.04	1
	6	Vanillic acid[#]	$C_8H_8O_4$	167.0344	167.0344	151.00, 123.04, 107.01	1
	7	Gallic acid[#]	$C_7H_6O_5$	169.0134	169.0137	125.02	1
	8	Ethyl protocatechuate	$C_9H_{10}O_4$	181.0501	181.05	137.06, 121.02, 107.04	2
	9	Methoxygallate[#]	$C_8H_8O_5$	183.029	183.0293	166.99, 139.04, 123.01, 111.01	1
	10	Ferulic acid	$C_{10}H_{10}O_4$	193.0502	193.05	177.01, 149.02, 133.02	1
	11	Syringic acid[#]	$C_9H_{10}O_5$	197.0451	197.045	181.01, 151.00, 137.02, 125.02	1
	12	Methyl ferulate	$C_{11}H_{12}O_4$	207.0658	207.0657	193.05, 177.05, 149.06, 135.04, 117.03	2
	13	5-Hydroxyferulic acid	$C_{10}H_{10}O_5$	209.045	209.045	191.03, 177.01, 165.05, 137.06, 123.00	2
	14	Propyl gallate	$C_{10}H_{12}O_5$	211.0606	211.0606	169.02, 125.00	2
	15	Carboxyvanillic acid	$C_9H_8O_6$	211.0246	211.0243	167.02, 151.00, 123.04, 107.01	2
	16	Aspidinol	$C_{12}H_{16}O_4$	223.0969	223.0970	209.08, 167.03, 149.02	2
	17	Sinapic acid[#]	$C_{11}H_{12}O_5$	223.0612	223.0606	208.03, 179.02, 164.00	1
	18	Chorismic acid	$C_{10}H_{10}O_6$	225.0399	225.0399	207.02, 181.05, 137.06	2
	19	Dihydrosinapic acid	$C_{11}H_{14}O_5$	225.0755	225.0763	209.04, 181.08, 163.07	2
	20	Trimethoxycinnamic acid	$C_{12}H_{14}O_5$	237.0765	237.0763	221.07, 206.07, 193.08, 178.06, 163.02	2
	21	Elenolic acid	$C_{11}H_{14}O_6$	241.0719	241.0712	209.04, 165.05, 139.04, 121.03	2
	22	Sulochrin	$C_{17}H_{16}O_7$	331.0823	331.0818	301.07, 181.05, 149.02	2
	23	Methylsulochrin	$C_{18}H_{18}O_7$	345.0979	345.0974	331.08, 301.07, 181.05, 149.02	3
	24	Ethylgallate-O-gallic acid	$C_{16}H_{14}O_9$	349.056	349.056	197.02, 169.01, 125.00	2
	25	Sulochrin sulphate[$]	$C_{17}H_{16}O_{10}S$	411.0389	411.0386	331.08, 303.08, 289.07	3
	26	Methylsulochrin sulphate	$C_{18}H_{18}O_{10}S$	425.0549	425.0542	344.02, 331.08, 301.07, 181.05, 149.02	3

	27	1,2-di-(syringoyl)-hexoside	$C_{24}H_{28}O_{14}$	539.1397	539.1401	359.09, 341.08, 197.04, 153.05	2
	28	Diferuloylquinic acid	$C_{27}H_{28}O_{12}$	543.1504	543.1503	499.16, 367.10, 323.11, 193.05	2
Flavonoids and derivatives	29	Naringenin#	$C_{15}H_{12}O_5$	271.0613	271.0606	187.03, 151.00, 135.00, 119.05	1
	30	Kaempferol#	$C_{15}H_{10}O_6$	285.0405	285.0399	213.02, 165.01, 151.00, 133.02,	1
	31	Aromadendrin#	$C_{15}H_{12}O_6$	287.0548	287.0561	285.04, 241.05, 177.01, 151.04	1
	32	Catechin/Epicatechin#	$C_{15}H_{14}O_6$	289.0692	289.0712	259.05, 243.07, 215.07	1
	33	Taxifolin	$C_{15}H_{12}O_7$	303.047	303.0504	285.04, 151.04, 137.02, 125.02	2
	34	Methlycatechin	$C_{16}H_{16}O_6$	303.0868	303.0862	289.07, 259.05, 243.07, 215.07	2
	35	Isorhamnetin#	$C_{16}H_{12}O_7$	315.05	315.0504	302.01, 285.04, 241.05, 151.04	1
	36	Cajanol	$C_{17}H_{16}O_6$	315.0874	315.08069	285.07, 177.05, 139.04	2
	37	Dihydroisorhamnetin	$C_{16}H_{14}O_7$	317.0667	317.0661	271.06, 187.03, 151.00, 135.00, 119.05	2
	38	Rhamnazin	$C_{17}H_{14}O_7$	329.0667	329.0661	299.05, 271.02, 167.03, 153.01, 123.00	2
	39	Taxifolin 7,4'-dimethyl ether	$C_{17}H_{16}O_7$	331.0822	331.0817	441.02, 423.03, 305.02, 125.00	2
	40	6-Methoxytaxifolin	$C_{16}H_{14}O_8$	333.0611	333.061	245.04, 205.05, 179.01, 151.04, 137.02	2
	41	Naringenin-7-sulfate	$C_{15}H_{12}O_8S$	351.0175	351.0174	245.04, 205.05, 179.01, 151.04, 137.02	2
	42	Kaempferol 3-O-sulfate	$C_{15}H_{10}O_9S$	364.9972	364.9967	341.03, 311.05, 283.02, 269.04	2
	43	Epicatechin 7-sulfate	$C_{15}H_{14}O_9S$	369.0284	369.028	288.07, 259.05, 243.07, 215.07	2
	44	Hesperetin 7-sulfate	$C_{16}H_{14}O_9S$	381.0284	381.028	301.03, 165.01, 149.02, 135.00	2
	45	Taxifolin sulfate	$C_{15}H_{12}O_{10}S$	383.0074	383.0073	303.05, 285.04, 241.05, 177.02, 125.02	2
	46	Methylepicatechin 3'-sulfate	$C_{16}H_{16}O_9S$	383.0442	383.0436	303.08, 287.05	2
	47	Isohamnetin 3-sulfate (Persicarin)	$C_{16}H_{12}O_{10}S$	395.0074	395.0072	318.02, 285.04, 241.05, 177.01, 151.04	2
	48	Cajanol Sulphate	$C_{17}H_{16}O_9S$	395.0439	395.043	314.04, 285.07, 177.05, 139.04	2
	49	Methyl taxifolin sulphate	$C_{16}H_{14}O_{10}S$	397.0233	397.0229	316.02, 285.04, 241.05, 177.01, 151.04	2
	50	Quercetin 7,3',4'-trimethyl ether Sulphate	$C_{18}H_{16}O_{10}S$	423.0391	423.0386	343.03, 314.05, 301.05, 135.02	3
	51	Kaempferol-3-O-rhamnoside (Afzelin) #	$C_{21}H_{20}O_{10}$	431.0984	431.0978	299, 289, 273, 258, 231, 207	1

	52	Eriodictyol glucoside	$C_{21}H_{22}O_{11}$	449.108	449.1083	287.14, 269.05, 251.03, 165.02	2
	53	Kaempferol 3-glucuronide#	$C_{21}H_{18}O_{12}$	461.0724	461.072	307.06, 289.05, 275.03, 241.05	1
	54	Dillanoside	$C_{24}H_{22}O_{10}$	469.1144	469.1135	169.01, 167.05, 140.02, 139.05, 125.00	2
	55	Quercetin-4'-glucuronide	$C_{21}H_{18}O_{13}$	477.067	477.0669	289.07, 273.08	2
	56	Eriodictyol 7-(6-galloylglucoside)	$C_{28}H_{26}O_{15}$	601.1196	601.1193	481.08, 439.06, 303.05, 167.03, 125.02	2
	57	Isorhamnetin 3-rutinoside	$C_{28}H_{32}O_{16}$	623.1618	623.1612	463.05, 300.99, 273.00, 179.03, 163.04	2
Tannins	58	Epicatechin-3-gallate	$C_{22}H_{18}O_{10}$	441.0824	441.0821	371.04, 289.05, 271.04, 179.01, 169.01, 125.02, 109.02	
	59	Epicatechin 3-(3-methylgallate)	$C_{23}H_{20}O_{10}$	455.0981	455.0978	305.04, 287.03, 169.01, 151.02, 125.00	2
	60	Gallocatechin gallate	$C_{22}H_{18}O_{11}$	457.0774	457.0771	285.03, 255.04, 227.05, 151.00	2
	61	Epigallocatechin 3-(3-methylgallate)	$C_{23}H_{20}O_{11}$	471.0931	471.0927	301.02, 273.02, 257.02, 151.00, 125.02	2
	62	Fisetinidol-(4α,8)-catechin	$C_{30}H_{26}O_{11}$	561.1404	561.1397	465.08, 439.06, 301.03, 289.07, 125.02	2
	63	Procyanidin A2#	$C_{30}H_{24}O_{12}$	575.1200	575.1189	451.10, 425.08, 407.07, 289.07, 287.05, 125.00	1
	64	Procyanidin B2#	$C_{30}H_{26}O_{12}$	577.1346	577.1352	300.05, 193.02, 165.03, 151.00, 121.07, 107.05	1
	65	Procyanidin A4	$C_{30}H_{24}O_{13}$	591.1143	591.1139	467.09, 441.08, 303.05, 289.07, 125.02	2
	66	Gallocatechin-catechin	$C_{30}H_{26}O_{13}$	593.1302	593.1295	313.05, 287.05, 269.01, 151.01, 125.00	2
	67	Prodelphinidin A1	$C_{30}H_{24}O_{14}$	607.1093	607.1088	477.10, 315.05, 299.05, 165.05	2
	68	Prodelphinidin B	$C_{30}H_{26}O_{14}$	609.1233	609.1244	271.07, 163.05	2
	69	Balanophotannin B	$C_{29}H_{22}O_{16}$	625.0839	625.083	441.08, 289.07, 169.01	2
	70	Catechin gallate derivatives	$C_{29}H_{30}O_{16}$	633.1461	633.1456	473.36, 443.35, 341.28, 289.21	2
	71	Bryonioside A	$C_{36}H_{60}O_{9}$	635.4162	635.4159	441.08, 407.00, 289.07, 169.01	2
	72	Procyanidin B2-gallate	$C_{37}H_{30}O_{16}$	729.1461	729.1455	739.05, 713.05, 695.21, 577.02, 451.05, 289.04,	2

	73	Procyanidin C1	$C_{45}H_{38}O_{18}$	865.1982	865.1979	729.14, 441.08, 407.00, 289.07, 169.01	2
	74	Procyanidin B2-digallate	$C_{44}H_{34}O_{20}$	881.1574	881.1565	187.31, 124.91, 169.20, 111.20	2
Coumarins	75	Aesculetin#	$C_9H_6O_4$	177.0194	177.0188	149.02, 133.02, 105.03	1
	76	Bergaptol	$C_{11}H_6O_4$	201.0188	201.0188	185.02, 157.17, 129.10	2
	77	Umbelliferone carboxylic acid	$C_{10}H_6O_5$	205.0088	205.0137	161.02, 133.02	2
	78	Citropten	$C_{11}H_{10}O_4$	205.0519	205.0501	191.02, 161.06, 133.02	2
	79	Methylumbelliferyl acetate	$C_{12}H_{10}O_4$	217.0503	217.05	175.03, 133.02	2
	80	6,7-Dihydroxycoumarin-3-carboxylic Acid	$C_{10}H_6O_6$	221.0121	221.0098	177.01, 133.02	2
	81	Fraxidin	$C_{11}H_{10}O_5$	221.045	221.045	207.02, 193.02, 123.02	1
	82	Dihydrocoriandrin	$C_{13}H_{12}O_4$	231.0657	231.0657	200.04, 159.08, 144.05	2
Carboxylic acid, fatty acids and amino acids	83	Ornithine	$C_5H_{12}N_2O_2$	131.0825	131.0821	114.07, 113.05, 68.06	2
	84	Malic acid	$C_4H_6O_5$	133.0124	133.0137	115.00, 89.02, 71.01	2
	85	Pelargonic acid	$C_9H_{18}O_2$	157.1222	157.1228	139.07, 113.05	2
	86	Hydroxyadipic acid	$C_6H_{10}O_5$	161.045	161.045	143.03, 101.02, 99.04	2
	87	Ribonic acid	$C_5H_{10}O_6$	165.0395	165.0399	149.04, 105.01, 87.00, 75.00	2
	88	L-Citrulline	$C_6H_{13}N_3O_3$	174.0898	174.0879	116.07, 99.04, 70.06	2
	89	Ascorbic acid	$C_6H_8O_6$	175.0243	175.0242	142.99, 115.00, 89.02	2
	90	Azelaic acid	$C_9H_{16}O_6$	187.0976	187.0969	187.31, 124.91, 169.20, 111.20	2
	91	Quinic acid#	$C_7H_{12}O_6$	191.0553	191.0555	173.05, 127.04, 93.03, 85.03	2
	92	Gluconic acid	$C_6H_{12}O_7$	195.0522	195.051	177.01. 151.06. 129.02. 121.04	2
	93	Homocitric acid	$C_7H_{10}O_7$	205.0351	205.0354	161.04. 143.04. 117.05	2
	94	Myristic acid	$C_{14}H_{28}O_2$	227.2017	227.2015	209.19, 183.21, 179.18	2
	95	N-undecanoylglycine	$C_{13}H_{25}NO_3$	242.1756	242.1756	224.16, 182.15	2
	96	Palmitic acid	$C_{16}H_{32}O_2$	255.233	255.233	237.23, 211.24, 197.22	2
	97	α-Linoleic acid	$C_{18}H_{32}O_2$	279.233	279.2331	261.22, 235.24, 233.22	2
	98	Oleic acid	$C_{18}H_{34}O_2$	281.2487	281.2486	263.25. 181.21. 127.25	2
	99	Stearic Acid	$C_{18}H_{36}O_2$	283.2643	283.2637	265.24. 239.25. 209.22. 183.19. 171.12	2

	100	Hydroxy octadecatrienoic acid	$C_{18}H_{30}O_3$	293.2122	293.2116	275.20. 223.03. 195.13. 183.13. 171.10	2
	101	Hydroxy linoleic acid	$C_{18}H_{32}O_3$	295.2312	295.2276	277.20. 253.02. 223.03. 167.05	2
	102	Ricinoleic acid	$C_{18}H_{34}O_3$	297.2434	297.243	279.23, 253.25, 235.24, 165.12	1
	103	Retinoic acid	$C_{20}H_{28}O_2$	299.2015	299.2011	283.16, 255.21, 239.18, 135.11	2
	104	Octadecendioic acid	$C_{18}H_{32}O_4$	311.2239	311.223	299.25, 269.24, 251.23, 233.24	2
	105	Trihydroxy-octadecadienoic acid	$C_{18}H_{32}O_5$	327.217	327.2171	299.12. 285.21. 229.14. 211.13. 171.10	2
	106	Pinellic acid	$C_{18}H_{24}O_5$	329.2329	329.2328	229.14. 211.13. 171.10	2
	107	Tricosanoic acid	$C_{23}H_{46}O_2$	353.342	353.3419	309.14, 241.09, 171.25	2
	108	Hydroxybehenic acid	$C_{22}H_{44}O_3$	355.3214	355.3212	337.31, 311.33, 293.32, 281.32	2
	109	Cerebronic acid	$C_{24}H_{48}O_3$	383.3531	383.3525	365.34, 339.36, 321.35, 309.35	3
Others	110	8-Hydroxy-(+)-δ-cadinene	$C_{15}H_{24}O$	219.175	219.1748	203.14, 201.16, 179.14	2
	111	Methyl Jasmonic acid	$C_{13}H_{20}O_3$	223.1336	223.1334	208.06, 193.11, 151.11, 133.09, 109.06	2
	112	Thymol sulphate	$C_{10}H_{14}O_4S$	229.0502	229.0501	149.09, 133.06	2
	113	Gomphidic acid	$C_{18}H_{12}O_9$	371.0405	371.0403	301.07, 285.03, 163.07, 150.00, 135.01	3
	114	Stictic acid	$C_{19}H_{14}O_9$	385.0563	385.056	367.04, 341.06, 311.05, 297.07, 267.06, 205.01	3
	115	Phloridzin	$C_{21}H_{24}O_{10}$	435.1294	435.1291	273.00, 229.10, 167.05, 123.05	1
	116	Betulinic acid#	$C_{30}H_{48}O_3$	455.3526	455.3525	437.34, 409.36, 393.35, 247.18, 207.19, 201.16, 189.16	1
	117	Resveratrol 3-glucoside 5-sulfate$	$C_{20}H_{22}O_{11}S$	469.0807	469.0805	389.12, 307.02, 289.17, 227.07	3
	118	Resveratrol 4'-(2"-galloylglucoside) $	$C_{27}H_{26}O_{12}$	541.135	541.1346	371.11, 227.07, 209.06, 169.01, 125.02	3

EF: elemental formula; OM: observed mass; CM: calculated mass; CL: confidence level; #: confirmed with reference standard; $: first time identified in DIB.
